# Bioactivities of the Genus *Combretum* (Combretaceae): A Review

**DOI:** 10.3390/molecules17089142

**Published:** 2012-08-02

**Authors:** Gedson Rodrigues de Morais Lima, Igor Rafael Praxedes de Sales, Marcelo Ricardo Dutra Caldas Filho, Neyres Zínia Taveira de Jesus, Heloina de Sousa Falcão, José Maria Barbosa-Filho, Analúcia Guedes Silveira Cabral, Augusto Lopes Souto, Josean Fechine Tavares, Leônia Maria Batista

**Affiliations:** Department of Pharmaceutical Sciences, Federal University of Paraiba, João Pessoa 58051-970, PB, Brazil; Email: gedson@ltf.ufpb.br (G.R.M.L.); igor_caraubas@hotmail.com (I.R.P.S.); marcelo.dutra@ltf.ufpb.br (M.R.D.C.F.); neyresj@hotmail.com (N.Z.T.J.); heloinafalcao@yahoo.com.br (H.S.F.); jbarbosa@ltf.ufpb.br (J.M.B.-F.); analuciaguedes@gmail.com (A.G.S.C.); augustosouto@gmail.com (A.L.S.); josean@ltf.ufpb.br (J.F.T.)

**Keywords:** Combretaceae, *Combretum*, bioactivity, medicinal plants, natural products, review

## Abstract

The Combretaceae is a large family of herbs, shrubs and trees, comprising about 20 genera and 600 species with tropical distribution around the globe and centers of diversity in Africa and Asia. Some *Combretum* species are extensively used in traditional medicine against inflammation, infections, diabetes, malaria, bleeding, diarrhea and digestive disorders and others as a diuretic. The present work is a literature survey of *Combretum* species that have been evaluated for their ability to exert biological activities. A total number of 36 *Combretum* species are discussed with regard to plant parts used, component tested and bioassay models. This review is of fundamental importance to promoting studies on *Combretum* species, thereby contributing to the development of new therapeutic alternatives that may improve the health of people suffering from various health problems.

## 1. Introduction

Medicinal plants have been used since ancient times in virtually all cultures as a source of medicines [1], and are of great importance to the health of individuals and communities [2]. Traditional medicine is used in all parts of the World and has a rapidly growing economic importance, mainly through the use of medicinal plants, especially in developing countries [3]. The medicinal use of plants of the family Combretaceae is widely described in the scientific literature [4,5,6]. This family is distributed in appoximately 20 genera with 600 species. The largest genera are *Combretum* and *Terminalia*, with about 370 and 200 species, respectively [7]. Members of the Combretaceae occur mainly in tropical and subtropical areas, for example, in Africa and Brazil.

### Phytochemical Components Isolated from the Active Combretum Species

**Figure 1:**
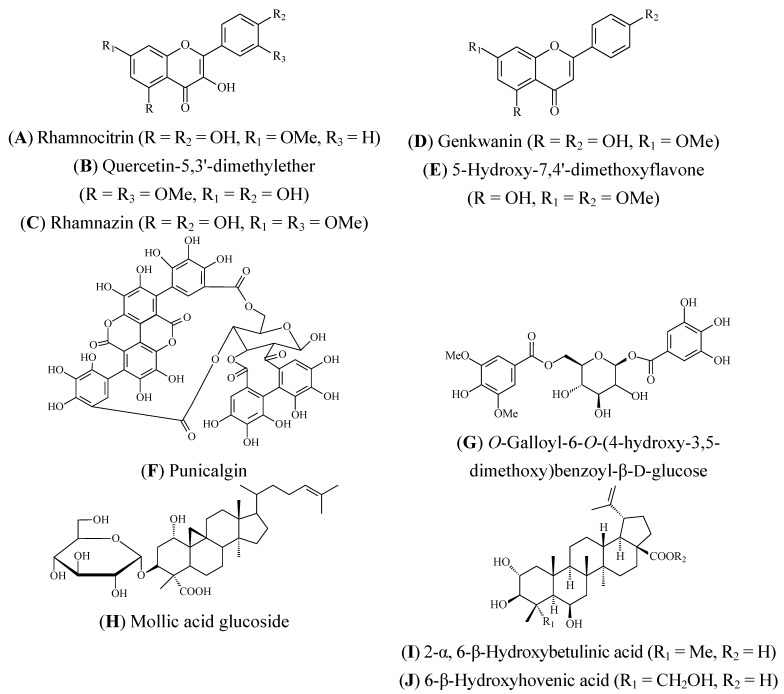
The molecular structures of compounds isolated from *Combretum* species.

Phytochemical studies carried out in the genus *Combretum* have demonstrated the occurrence of many classes of constituents, including triterpenes, flavonoids, lignans and non-protein amino acids, among others [7]. Since the 1970s, several unusual compounds have also been isolated from *Combretum* species, for example, 9,10-dihydrophenanthrenes and a substituted bibenzyl from *C. molle* [8]. Bisoli *et al.* isolated 11 triterpenes and their glycosides from *C. laxum*, among them, oleanane-, ursane- and lupane-type such as arjunolic acid, arjunglucoside II, bellericoside, chebuloside II, quadranoside IV, asiatic acid and betulinic acid [9]. Cycloartane dienone lactone was isolated from *C. quadrangulare* [10], and alkaloids (combretine and betonicine) from the leaves of *C. micranthum* [11]. Some flavonoids, rhamnoctrin (Figure 1A), quercetin-5,3'-dimetylether (Figure 1B), ramnazin (Figure 1C) and kaempferol were isolated from *C. erythrophyllum* [12], as well as quercetrin, kaempferol and pinocembrin (flavanone) from *C. apiculatum* [13]. Cardamonin (chalcone) was also isolated from *C. apiculatum* [13] and ellagic acid derivatives from *C. kraussii *[14]. Combretastatins, a group of stilbenes, have been isolated from several species of *Combretum* [15].

As referenced above, there are several studies describing the phytochemistry of the species of this family, and the medicinal value of plants lies in the chemical substances that produce a physiological change in the human body [2]. Therefore, in continuation of our research on bioactive molecules from the various species of different plant families [16,17,18,19,20,21,22,23,24,25,26,27,28,29,30,31,32,33,34,35,36,37,38,39,40,41,42,43,44,45,46,47], the aim of this study was to review the literature on the bioactivity of the genus *Combretum*.

## 2. Results and Discussion

In this review, it was possible to list thirty-six species of the genus *Combretum*. The effectiveness of the plant extracts depended on the type of drug studied and the bioassay models. Thus, it was possible to classify the extracts as active or inactive. In this study, we chose more species referenced in data collected in the NAPRALERT natural products database and the scientific literature databases ScienceDirect and PubMed. 

*Combretum micranthum* is a bushy shrub or creeper found all over Africa. *C. micranthum* is used in traditional medicine for the treatment of wounds and sores [48,49,50] and of fever (especially malaria fever), cough and bronchitis [49,51]. In studies evaluating its antibacterial activity, the extracts used were obtained with different solvents (ethanol, chloroform, methanol or water). Activity was observed against the following bacterial species: *Pseudomonas aeruginosa, Staphylococcus aureus, Salmonella* species, *Streptococcus* species, *Proteus vulgaris*, *Klebsiella* species, *Sarcina lutea*, *Micrococcus luteus* and *Bacillus subtilis* [52,53,54,55,56,57]. In addition, antifungal activity against *Candida albicans* was noted [56]. Antiviral activity of a methanolic extract was reported against *Herpes simplex 1* and *Herpes simplex 2* [58]. Toxicity studies have reported the activity of an ethanolic extract in the brine shrimp lethality test [56]. Benoit *et al.* [59] and Karou *et al.* [60] reported anti-Malarial activity against *Plasmodium falciparum*. However, a methanolic extract did not display cytotoxic activity aganist THP1 cells [61] (Table 1). 

Di Carlo *et al.* [62] demonstrated immuno-stimulating activity with a suspension of powdered leaf. Chika and Bello [63] demonstrated an antidiabetic effect for the aqueous leaf extract of *C. micranthum*. A dose of 100 mg/kg of the extract was the most effective, among the doses tested. It produced a significant hypoglycemic and antidiabetic activity comparable to the effect of a standard drug (0.6 mg/kg glibenclamide) (Table 1). This study demonstrated the potential antidiabetic properties of aqueous leaf extract of *C. micranthum* for both type 1 and type 2 diabetes, justifying its traditional use in the treatment of this disease in Northwestern Nigeria. All of the above results contribute to justifying the use of the plant in traditional medicine for treating various conditions, particularly infections and diabetes. 

*C. molle* (soft-leaved *Combretum*, velvet bush willow) is a tree with a larger, straighter trunk compared to most species of *Combretum*, further distinguished by its rough bark and dense crown. It occurs throughout tropical Africa and in the Arabian Peninsula in areas where woodlands and wooded grasslands predominate, often forming pure stands on hillsides [64].

*C. molle* has been widely used as a medicinal plant to treat various diseases such as parasitic, protozoan and other infectious diseases in East [65,66,67] and West Africa [68]. Antibacterial studies have demonstrated its activity against *Staphylococcus aureus* and *Helicobacter pylori* at different extract concentrations [69,70,71]. Antifungal activity was reported in models that used *Epidermophyton floccosum*, *Microsporum gypseum*, *Trichophyton mentagrophytes*, *T. rubrum*, *Candida albicans*, *C. neoformans*, *Aspergillus fumigatus*, *Sporothrix schenckii* and *Microsporum canis* [72,73]. *C. molle* was also able to inhibit the growth of *Mycobacterium tuberculosis* [74]. Antitrypansomal and anthelmintic activities of different extracts have also been reported [4,75,76,77] (Table 1).

Toxicity studies have reported the activity of aqueous and acetone extracts against *Artemia salina* [9]. Furthermore, Asres *et al.* [78] and Gansané * et al.* [6] reported antimalarial activity of the methanolic extract against *Plasmodium falciparum* at different concentrations tested. Molluscicidal effect of aqueous extract against *Biomphalaria pfeifferi* was also observed [75]. Meanwhile, embryotoxic effects have not been reported [79] (Table 1).

Methanolic extracts of the roots and leaves (25 μg/mL) of *C. molle* showed strong cytotoxic effects against T-24 bladder cancer cells [15]. In addition, the aqueous and methanol extracts of *C. molle* were screened for inhibitory effects against HIV-1 reverse transcriptase. These extracts produced relatively strong inhibition of RNA-dependent-DNA polymerase (RDDP) activity. The compounds responsible for these activities in this plant were not sought [80] (Table 1).

In the case of compounds obtained from *C. molle*, the analgesic and antiinflammatory properties of mollic acid glucoside (MAG) (Figure 1H), a 1α-hydroxycycloartenoid extracted from *Combretum molle* leaves, have been investigated in mice and rats [81]. The results of this laboratory animal study indicate that MAG possesses analgesic and antiinflammatory effects in the mammalian models used. The author suggested that MAG possesses both centrally- and peripherally-mediated analgesic effects. 

Ojewole also reported on the cardiovascular effects of MAG. The results of this study showed that this compound was capable of causing bradycardia, vasorelaxation and hypotension in the animals evaluated [82]. In addition, hypoglycemic and antidiabetic activity have also been demonstrated [83].

*In vitro* anti-HIV activity of two isolated tannins from an acetone fraction, punicalgin (Figure 1F) and CM-A (whose structure has not yet been fully elucidated), was assessed against human immunodeficiency virus type 1 (HIV-1) and type 2 (HIV-2). The results displayed selective inhibition of HIV-1 replication with selective indices (ratio of 50% cytotoxic concentration to 50% effective antiviral concentration) of 16 and 25, respectively and afforded complete cell protection against the virus-induced cytopathic effect when compared to control samples. Neither of the tannins was able to inhibit HIV-2 replication [84].

These results contribute to the validation of the popular use of this plant species in the treatment of bacterial, fungal, protozoan and viral infections and cardiovascular problems, among others.

The plant *C. erythrophyllum* (Burch.) Sond., commonly known as river *Combretum*, is a medium-sized, spreading, densely foliaged tree up to 12 m in height, which has been used by traditional healers for a variety of disorders [85,86]. *C. erythrophyllum* is widely used in traditional medical practice in southern Africa. It has been used for treating abdominal pains and venereal diseases, which suggests the presence of antibacterial compounds in the leaves [87].

As part of the treatment for venereal diseases, powdered roots of *C. erythrophyllum* are inserted into the vagina, which has resulted in several fatalities. The same procedure is followed to reduce the size of the vaginal orifice. In addition, the plant has been used to treat sexually transmitted diseases [85].

Extracts of *C. erythrophyllum* obtained with different solvents (acetone, hexane, chloroform, carbon tetrachloride and butanol) have shown antibacterial activity at different doses against *Escherichia coli*, *Pseudomonas aeruginosa*, *Staphylococcus aureus *and *Enterococcus faecalis *[88,89] (Table 1). Some antibacterial flavonoids were subsequently isolated by bioassay-guided fractionation, namely genkwanin (Figure 1D), 5-hydroxy-7,4-dimethoxyflavone (Figure 1E), rhamnocitrin (Figure 1A), quercetin-5,3-dimethylether (Figure 1B), and rhamnazin (Figure 1C). These compounds showed good activity against *Micrococcus luteus*, *Shigella sonei*, *Vibrio cholerae*, *Enterococcus faecalis* and *Pseudomonas aeruginosa*. The results provide a clear rationale for the ethnomedicinal use of *C. erythrophyllum* leaves in treating bacterial infections [12]. Furthermore, these compounds have demonstrated antiinflammatory activity in experimental models *in vitro* [12]. 

Moreover, in studies evaluating antifungal activity, extracts obtained with different solvents (acetone, hexane, dichloromethane and methanol) were active against the following species: *C. albicans*, *C. neoformans*, *A. fumigatus*, *S. schenckii *and *M. canis* [73] (Table 1).

Toxicity studies have shown that the aqueous extract of *C. erythrophyllum* has mutagenic activity against *Salmonella typhimurium* [90]. The aqueous extract causes mutations in the meiotic stage of *Drosophila melanogaster* [86]. The methanol, dichloromethane and acetate extracts of *C. erythrophyllum* showed bioactivity in a yeast-based microtiter assay for DNA-damaging agents [91] (Table 1).

*C. erythrophyllum* extract has spasmolytic activity in the pre-contracted uterus, and this activity seems to involve the inhibition of cyclooxygenase, blocking the biosynthesis of prostaglandins, substances that are involved in uterine muscle contraction [92].

The alcoholic extract of *Combretum dolichopetalum* is used in folklore medicine to relieve stomach ache, blood in the stools, diarrhea, cramps and related gastrointestinal disorders [93]. The ethanolic extract of *C. dolichopetalum* has shown a gastroprotective effect in stress-induced and non-steroidal antiinflammatory (indomethacin)-induced ulcer models. The crude extract inhibited secretions induced in rats by pyloric ligation together with histamine [93,94] (Table 1). In addition, the pharmacological actions were evaluated in the guinea-pig isolated ileum and in intact rats. The crude extract inhibited the contractions induced by acetylcholine and histamine in the guinea-pig ileum in a concentration-dependent manner. The extract also delayed gastric emptying in rats in a dose-dependent manner. These results therefore suggest that *C. dolichopetalum* has gastric antisecretory activity, increasing gastric emptying time, and acts as a smooth muscle relaxant and spasmolytic agent [93,94] (Table 1).

The hepatoprotective effects of the ethanolic extract of *C. dolichopetalum* root bark were evaluated on paracetamol-induced liver intoxication in rats. Oral pre-treatment with *C. dolichopetalum* ethanolic extract significantly attenuated the elevation of serum glutamate-oxaloacetate transaminase (GOT) and glutamate- pyruvate transaminase (GPT) induced by paracetamol intoxication in rats [95] (Table 1).

Asuzu *et al. *[94] demonstrated that the methanol and chloroform extracts obtained with dried roots of *C. dolichopetalum* have antiinflammatory activity in models of carrageenan-induced paw edema and croton oil-induced edema in mice [96]. Udem *et al.* conducted toxicity studies in rats and found activity in both sexes (LD_50_ 246.0 mg/kg) [97] (Table 1).

*Combretum quadrangulare* is a shrub or tree, indigenous to southeast Asia, especially Burma to Laos. The plant is commonly known as “tram bâu” (Vietnam), “kê khao” (Laos) or “sang kaê” (Cambodia), and the seeds are used in Vietnamese traditional medicine as a remedy against round and tapeworm infections in humans [98]. Studies conducted by Somanabandhu *et al.* [99] revealed the ether and ethanolic extracts of dried root bark or dried seed are effective against earthworms when tested *in vitro* [99]. Antimicrobial activity was also reported in extracts of dried leaves, which were active against *Helicobacter pylori* [100] (Table 1).

The hepatoprotective effect of MeOH, MeOH/H_2_O (1:1) and aqueous extracts of *C. quadrangulare* seeds were examined on D-galactosamine (D-GalN)/tumor necrosis factor-α (TNF-α)-induced cell death in primary cultured mouse hepatocytes. The MeOH extract showed the strongest inhibitory effect on D-GalN/TNF-α-induced cell death (IC_50_ 56.4 μg/mL). Moreover, the MeOH extract also significantly lowered the serum GPT level in mice with D-GalN/lipopolysaccharide (LPS)-induced liver injury [101] (Table 1). Acetone, MeOH, and aqueous extracts of *C. quadrangulare* were tested for their trypanocidal activity against epimastigotes of *Trypanosoma cruzi*, the causative agent of Chagas disease. Strong trypanocidal activity was found in the acetone extract of *C. quadrangulare* [102] (Table 1). 

The aqueous and EtOH extracts of *C. quadrangulare* were screened for their inhibitory activity against HIV-1 integrase (IN), an enzyme essential for viral replication. The aqueous and EtOH extracts showed significant inhibitory activity against HIV-1 with an IC_50_ value of 2.5 and 2.9 µg/mL, respectively [103] (Table 1). The compound *O*-galloyl-6-*O*-(4-hydroxy-3,5-dimethoxy)benzoyl-*β*-D-glucose (Figure 1G), a new gallic acid derivative isolated from *C. quadrangulare*, demonstrated potent hepatoprotective activity against D-GalN/TNF-alpha-induced cell death in primary cultured mouse hepatocytes [104]. The triterpenes of the lupane type, 2*α*,6*β*-dihydroxybetulinic acid (Figure 1I) and 6*β*-hydroxyhovenic acid (Figure 1J), isolated from the MeOH extract of *C. quadrangulare* seeds, also exhibited strong hepatoprotective activity [105].

## 3. Material and Methods

The biological activity of the *Combretum* species was searched through the NAPRALERT (acronym for Natural Products ALERT) databank of the University of Illinois at Chicago. The data were updated in April 2011, using biological activity of the *Combretum* species as search term. The plant extracts were selected for this work and the references found in the search were later consulted for details on the models or mechanisms. Furthermore, this data survey was supplemented with searches in the PubMed and ScienceDirect sites. The specific names of the species were used as keywords.

**Table 1:** Bioactivities of drugs obtained of the genus botanical *Combretum*.

Biological Activity	Botanical Name	Part Tested	Bioassay Models	Result
*Enzymatic activity*				
Inhibition of acetylcholinesterase				
	*C. hartmannianum* Schweinf.	MeCl_2_ or AcOEt ext. of dried leaf	*In vitro-*TLC and Microplate assay by Ellman’s method	Inactive [106]
		EtOH ext. of dried leaf	*In vitro-*TLC and Microplate assay by Ellman’s method-IC_50_ for drug: 0.25 mg/mL	Active [106]
		MeCl_2_ and EtOH ext. of dried stem bark	*In vitro-*TLC and Microplate assay by Ellman’s method-IC_50_ for drugs: 1.0 or 0.37 mg/mL, respectively	Active [106]
		AcOEt ext. of dried stem bark	*In vitro-*TLC and Microplate assay	Inactive [106]
		EtOH ext. of dried root	*In vitro-*TLC and Micro-plate assay by Ellman’s method-IC_50_ for drug: 0.37 mg/mL	Active [106]
		MeCl_2_ and AcOEt ext. of dried root	*In vitro-*TLC and Microplate assay by Ellman’s method	Inactive [106]
Inhibition of ACE				
	*C. fruticosum *(Loefl.) Stuntz	MeOH/MeCl_2_ (50:50) ext. of dried stem or dried leaf	*In vitro*-ACE isolated from rabbit lung catalyze the cleavage of the chromophore-fluorophore-labeled substract dansyltriglycine into dansylglycine—Concentration for drugs: 0.33 mg/mL	Active [107]
		EtOH ext. of leaves	*In vitro*-ACE isolated from rabbit lung catalyze the cleavage of hippuryl-glycyl-glycine and react with trinitrobenzenesulfonic acid to form 2,4,6-trinitrophenyl glycyl glycine—Concentration for drug: 0.33 mg/mL	Active [108,109]
Inhibition of topoisomerase				
	*C. apiculatum* Sond. subsp *apiculatum*	EtOAc ext. of dried rootbark, or dried stemwood, or dried rootwood	*In vitro*-Topoisomerase I or topoisomerase II inhibition assay after Polyvinylpyrrolidine (1:1) or Collagen (1:100) methods, respectively	Active [110]
	*C. erythrophyllum* (Burch.) Sond.	EtOAc ext. of dried leaf	*In vitro*-Topoisomerase I or topoisomerase II inhibition assay after Polyvinylpyrrolidine (1:1) or Collagen (1:100) methods, respectively	Active [110]
*Antiparasitic activity*				
Antiascariasis				
	*C. quadrangulare* Kurz.	Ether and EtOH (95%) ext. of dried root bark or dried seed	*In vitro-*Earthworms—Concentration not cited	Active [99]
Antifilariasis				
	*C. mucronatum* Schumach.	Hot H_2_O ext. of root	88 human adult infected with guinea worms—Dose for drug: 0.03 mg/kg (*p.o.*)	Active [111]
Anthelmintic				
	*C*. *apiculatum *Sond. subsp. *apiculatum*	H_2_O, Acetone and AcOEt ext. of dried leaf	*In vitro-*Worms of *Caenorhabditis elegans *var. Bristol—Concentration for drugs: 0.5 and 1 mg/mL	Active [112]
	*C*. *bracteosum *(Hochst.) Brandis ex Engl.	H_2_O, Acetone and AcOEt ext. of dried leaf	*In vitro-*Worms of *C. elegans *var. Bristol—Concentration for drugs: 0.5 and 1 mg/mL	Inactive [112]
	*C*. *celastroides *Welw ex Laws subsp. *celastroides*	H_2_O, Acetone and AcOEt ext. of dried leaf	*In vitro-*Worms of *C. elegans *var. Bristol—Concentration for drugs: 0.5 and 1 mg/mL	Inactive [112]
	*C*. *collinum *Fresen. subsp. *suluense *(Engl. & Diels) Okafor	H_2_O and AcOEt ext. of dried leaf	*In vitro-*Worms of *C. elegans *var. Bristol—Concentration for drugs: 0.5 and 1 mg/mL	Inactive [112]
		Acetone ext. of dried leaf	*In vitro-*Worms of *C. elegans *var. Bristol—Concentration for drugs: 0.5 and 1 mg/mL	Active [112]
	*C*. *edwardsii *Exell	H_2_O, Acetone and AcOEt ext. of dried leaf	*In vitro-*Worms of *C. elegans *var. Bristol—Concentration for drugs: 0.5 mg/mL	Inactive [112]
		Acetone and AcOEt ext. of dried leaf	*In vitro-*Worms of *C. elegans *var. Bristol—Concentration for drugs: 1 mg/mL	Active [112]
	*C*. *erythrophyllum *(Burch.) Sond.	H_2_O, Acetone and AcOEt ext. of dried leaf	*In vitro-*Worms of *C. elegans *var. Bristol—Concentration for drugs: 0.5 mg/mL	Inactive [112]
		Acetone and AcOEt ext. of dried leaf	*In vitro-*Worms of *C. elegans *var. Bristol—Concentration for drugs: 1 mg/mL	Active [112]
	*C*. *hereroense *Schinz	H_2_O, Acetone and AcOEt ext. of dried leaf	*In vitro-*Worms of *C. elegans *var. Bristol—Concentration for drugs: 0.5 mg/mL	Inactive [112]
		Acetone and AcOEt ext. of dried leaf	*In vitro-*Worms of *C. elegans *var. Bristol—Concentration for drugs: 1 mg/mL	Active [112]
	*C*. *imberbe *Wawra	H_2_O and AcOEt ext. of dried leaf	*In vitro-*Worms of *C. elegans *var. Bristol—Concentration for drugs: 0.5 and 1 mg/mL	Inactive [112]
		Acetone ext. of dried leaf	*In vitro-*Worms of *C. elegans *var. Bristol—Concentration for drugs: 1 mg/mL	Active [112]
	*C*. *kraussii *Hochst.	H_2_O and AcOEt ext. of dried leaf	*In vitro-*Worms of *C. elegans *var. Bristol—Concentration for drugs: 0.5 and 1 mg/mL	Inactive [112]
		Acetone ext. of dried leaf	*In vitro-*Worms of *C. elegans *var. Bristol—Concentration for drugs: 0.5 and 1 mg/mL	Active [112]
	*C*. *microphyllum *Klotzsch	H_2_O and AcOEt ext. of dried leaf	*In vitro-*Worms of *C. elegans *var. Bristol—Concentration for drugs: 0.5 and 1 mg/mL	Inactive [112]
		Acetone ext. of dried leaf	*In vitro-*Worms of *C. elegans *var. Bristol—Concentration for drugs: 0.5 and 1 mg/mL	Active [112]
	*C*. *mkuzense *Carr & Retief	H_2_O and AcOEt ext. of dried leaf	*In vitro-*Worms of *C. elegans *var. Bristol—Concentration for drugs: 0.5 and 1 mg/mL	Inactive [112]
		Acetone ext. of dried leaf	*In vitro-*Worms of *C. elegans *var. Bristol—Concentration for drugs: 0.5 and 1 mg/mL	Active [112]
	*C*. *moggii *Exell	H_2_O, Acetone and AcOEt ext. of dried leaf	*In vitro-*Worms of *C. elegans *var. Bristol—Concentration for drugs: 0.5 and 1 mg/mL	Inactive [112]
	*C*. *molle *R. Br. ex G. Don	H_2_O and AcOEt ext. of dried leaf	*In vitro-*Worms of *C. elegans *var. Bristol—Concentration for drugs: 0.5 and 1 mg/mL	Inactive [112]
		Acetone ext. of dried leaf	*In vitro-*Worms of *C. elegans *var. Bristol—Concentration for drugs: 0.5 and 1 mg/mL	Active [112]
		Acetone ext., n-butanol, hexane, CHCl_3_ or H_2_O/MeOH fractions of leaf	*In vitro*-Nematocidal activity by means of an egg hatch and larval development of *Haemonchus contortus*-Lethal Concentration 50% (LC_50_) for drugs: 0.866, 0.333, 0.833, 0.747 or 0.065 mg/mL, respectively	Active [77]
			*In vitro*-Nematocidal activity by means of an egg hatch and larval development of *Haemonchus contortus*-Lethal Concentration 50% (LC_50_) for drugs: 0.604, 0.362, 1.077, 0.131 or 0.318 mg/mL, respectively	Active [77]
		H_2_O/MeOH ext.	Lambs infected with larvae of *H. contortus*—Dose for drug: 500, 1,000 or 2,000 mg/kg (*p.o.*)	Active [113]
	*C*. *mossambicense *(Klotzsch) Engl.	H_2_O, Acetone and AcOEt ext. of dried leaf	*In vitro-*Worms of *C. elegans *var. Bristol—Concentration for drugs: 0.5 mg/mL	Inactive [112]
		Acetone and AcOEt ext. of dried leaf	*In vitro-*Worms of *C. elegans *var. Bristol—Concentration of drugs: 1 mg/mL	Active [112]
	*C. mucronatum *Schumach.	Hot H_2_O ext	Human adult infected with guinea worms—Dose not cited: (*p.o.*)	Active [114]
	*C*. *nelsonii *Dümmer	H_2_O and AcOEt ext. of dried leaf	*In vitro-*Worms of *C. elegans *var. Bristol—Concentration for drugs: 0.5 and 1 mg/mL	Inactive [112]
		Acetone ext. of dried leaf	*In vitro-*Worms of *C. elegans *var. Bristol—Concentration for drugs: 0.5 and 1 mg/mL	Active [112]
	*C. nigricans* Lepr.	MeCl_2_/MeOH ext. or H_2_O ext. of fruit	Motility warms of *Caenorhabditis elegans* Bristol—Concentration for drugs: 1 mg/mL	Active [115]
	*C*. *padoides *Engl. & Diels	H_2_O, Acetone and AcOEt ext. of dried leaf	*In vitro-*Worms of *C. elegans *var. Bristol—Concentration for drugs: 0.5 and 1 mg/mL	Inactive [112]
	*C*. *paniculatum *Vent.	H_2_O and AcOEt ext. of dried leaf	*In vitro-*Worms of *C. elegans *var. Bristol—Concentration for drugs: 0.5 and 1 mg/mL	Inactive [112]
		Acetone ext. of dried leaf	*In vitro-*Worms of *C. elegans *var. BristolConcentration for drugs: 0.5 and 1 mg/mL	Active [112]
	*C*. *petrophilum *Retief	H_2_O and AcOEt ext. of dried leaf	*In vitro-*Worms of *C. elegans *var. Bristol—Concentration for drugs: 0.5 and 1 mg/mL	Inactive [112]
		Acetone ext. of dried leaf	*In vitro-*Worms of *C. elegans *var. Bristol—Concentration for drugs: 0.5 and 1 mg/mL	Active [112]
	*C*. *woodii *Dümmer	H_2_O, Acetone and AcOEt ext. of dried leaf	*In vitro-*Worms of *C. elegans *var. Bristol—Concentration for drugs: 0.5 and 1 mg/mL	Inactive [112]
	*C*. *zeyheri *Sond.	H_2_O, Acetone and AcOEt ext. of dried leaf	*In vitro-*Worms of *C. elegans *var. Bristol—Concentration for drugs: 0.5 and 1 mg/mL	Inactive [112]
Antileishmaniasis				
	*C. comosum *G. Don.	MeOH, MeOH/H_2_O (50:50) or MeCl_2 _ext. of dried leaves	*In vitro*-Promastigotes of *Leishmania infantum*—Concentration for all drugs: >100.0 μg/mL	Inactive [116]
	*C. cuspidatum* Planch. ex Benth.	MeOH, MeOH/H_2_O (50:50) or MeCl_2 _ext. of dried leaves	*In vitro*-Promastigotes of *L. infantum*—Concentration for drugs: 34.5, >100.0 or 43.5 μg/mL, respectively	Inactive [116]
		MeOH, MeOH/H_2_O (50:50) or MeCl_2 _ext. of stem barks	*In vitro*-Promastigotes of *L. infantum*—Concentration for drugs: >100.0, >100.0 or 28.6 μg/mL, respectively	Inactive [116]
	*C. molle* R. Br. ex G. Don	Acetone fraction of stembark	*In vitro*—Murine peritoneal macrophages infected with amastigotes of *L. donovani*—Concentration for drug: 30.0 μg/mL	Inactive [76]
Antimalarial				
	*C. micranthum *G. Don.	EtOH (95%) ext. of dried leaf	*In vitro*-Cell culture (erythrocytes) with parasite maturation of *Plasmodium falciparum*—IC_50_ for drug: 33.05 μg/mL	Active [60]
		MeOH ext. of dried leaf	*In vitro*-Cell culture (*P. falciparum* W2)—Concentration for drug: >25 μg/mL	Inactive [61]
		Decoction or infusion of dried leaf and stem	*In vitro*-Cell culture (*P. falciparum* FcB1-Colombia chloroquine resistant)—IC_50_ for drug: 1.18 μg/mL	Active [59]
		Infusion of dried leaf and stem	*In vitro*-Cell culture (*P. falciparum* F32-Tanzania chloroquine-sensitive)—IC_50_ for drug: 1.7 μg/mL	Active [59]
		Decoction of dried leaf and stem	*In vitro*-Cell culture (*P. falciparum* F32-Tanzania chloroquine-sensitive)—IC_50_ for drug: 0.88 μg/mL	Active [59]
	*C. molle* R. Br. ex G. Don	Acetone fraction of stem bark	*In vitro*-Cell culture (Trophozoites of *P. falciparum*)—IC_50_ for drug: 8.17 μg/mL	Active [76]
		MeOH ext. of dried stem	*In vitro-*Cell culture with *P. falciparum*—IC_50_ for drug: 1.25 μg/mL	Active [78]
		EtOH (90%) ext. of leaves, rootbark or stembark	*In vitro-*Cell culture with *P. falciparum* K1—IC_50_ for drugs: 4.0 μg/mL	Active [4]
		MeOH or MeOH/H_2_O ext. of leaves	*In vitro*-Cell culture (K562S human monocyte infected with *P. falciparum* W2)-IC_50 _for drugs: 5.7 or 7.9 μg/mL, respectively	Active [6]
	*C. aff. psidioides* Welw. subsp*.psilophyllum *Wickens	EtOH (95%), Pet ether, EtOAc or H_2_O ext. of dried root bark	*In vitro*—Microdiluition assay (*P. falciparum*)—IC_50_ for drugs: 31.0, 39.0, 6.5or 30.0 μg/mL, respectively	Active [117]
	*C. racemosum* P. Beauv	EtOH (90%) ext. of leaves or root bark	*In vitro-*Cell culture with *P. falciparum* K1—IC_50_ for drug: 4.0 μg/mL	Active [4]
	*C. zeyheri *Sond.	MeCl_2_/MeOH (1:1) or H_2_O ext. of twigs	*In vitro*—Microdiluition assay (*P. falciparum *D10)—Concentration for drug: 15 or >100 μg/mL, respectively	Inactive [118]
Antischistosomal				
	*C. aculeatum* Vent.	H_2_O ext. of dried leaf	*In vitro*—Miracidicidal and cercaricidal activity on *Schistosoma mansoni*—Concentration for drug: 1,000 ppm	Active [119]
	*C*. *apiculatum *Sond. subsp. *apiculatum*	H_2_O ext. of dried leaf	*In vitro-*Worms of *Schistosoma haematobium*—Concentration not cited	Inactive [112]
	*C*. *bracteosum *(Hochst.) Brandis ex Engl.	H_2_O ext. of dried leaf	*In vitro-*Worms of *S. haematobium*—Concentration not cited	Inactive [112]
	*C*. *celastroides *Welw ex Laws subsp. *celastroides*	H_2_O ext. of dried leaf	*In vitro-*Worms of *S. haematobium*—Concentration not cited	Inactive [112]
			*In vitro*—Miracidicidal and cercaricidal activity on *S. mansoni*—Concentration for drug: 1,000 ppm	Active [119]
	*C*. *edwardsii *Exell	H_2_O ext. of dried leaf	*In vitro-*Worms of *S. haematobium*—Concentration not cited	Inactive [112]
	*C*. *erythrophyllum *(Burch.) Sond.	H_2_O ext. of dried leaf	*In vitro-*Worms of *S. haematobium*—Concentration not cited	Inactive [112]
	*C. glutinosum* Perrot. ex DC	H_2_O ext. of dried leaf	*In vitro*-Miracidicidal and cercaricidal activity on *S. mansoni*-Concentration for drug: 1,000 ppm	Active [119]
	*C. hartmannianum* Schweinf.	H_2_O ext. of dried leaf	*In vitro*-Miracidicidal and cercaricidal activity on *S. mansoni*-Concentration for drug: 1,000 ppm	Active [119]
	*C*. *hereroense *Schinz	H_2_O ext. of dried leaf	*In vitro-*Worms of *S. haematobium*-Concentration not cited	Inactive [112]
	*C*. *imberbe *Wawra	H_2_O ext. of dried root or dried leaf	*In vitro-*Worms of *S. haematobium*—MIC for drugs: 25.0 or 12.5 mg/mL, respectively	Active [112,120]
	*C*. *kraussii *Hochst.	H_2_O ext. of dried leaf	*In vitro-*Worms of *S. haematobium*—MIC for drug: 12.5 mg/mL	Active [112]
	*C*. *microphyllum *Klotzsch	H_2_O ext. of dried leaf	*In vitro-*Worms of *S. haematobium*—Concentration not cited	Inactive [112]
	*C*. *mkuzense *Carr & Retief	H_2_O ext. of dried leaf	*In vitro-*Worms of *S. haematobium*—Concentration not cited	Inactive [112]
	*C*. *moggii *Exell	H_2_O ext. of dried leaf	*In vitro-*Worms of *S. haematobium*—Concentration not cited	Inactive [112]
	*C*. *molle *R. Br. ex G. Don	H_2_O ext. of dried leaf	*In vitro-*Worms of *S. haematobium*—MIC for drug: 25 mg/mL	Active [112]
	*C*. *mossambicense *(Klotzsch) Engl.	H_2_O ext. of dried leaf	*In vitro-*Worms of *S. haematobium*—Concentration not cited	Inactive [112]
	*C*. *nelsonii *Dümmer	H_2_O ext. of dried leaf	*In vitro-*Worms of *S. haematobium*—MIC for drug: 12.5 mg/mL	Active [112]
	*C*. *padoides *Engl. & Diels	H_2_O ext. of dried leaf	*In vitro-*Worms of *S. haematobium*—Concentration not cited	Inactive [112]
	*C*. *paniculatum *Vent.	H_2_O ext. of dried leaf	*In vitro-*Worms of *S. haematobium*—MIC for drug: 25 mg/mL	Active [112]
	*C*. *petrophilum *Retief	H_2_O ext. of dried leaf	*In vitro-*Worms of *S. haematobium*—MIC for drug: 25 mg/mL	Active [112]
	*C*. *woodii *Dümmer	H_2_O ext. of dried leaf	*In vitro-*Worms of *S. haematobium*—Concentration not cited	Inactive [112]
	*C*. *zeyheri *Sond.	H_2_O ext. of dried leaf	*In vitro-*Worms of *S. haematobium*—Concentration not cited	Inactive [112]
Antitrypanosomal				
	*C. dolichopetalum *Gils ex Engl.	EtOH (70%) ext. of dried root bark	Infection induced in rats (*Trypanosoma brucei *or *Trypanosoma congolense* )—Dose for drug: 80.0 mg/kg (*i.p.*)	Active [97]
	*C. molle* R. Br. ex G. Don	Acetone fraction of stem bark	*In vitro*—Murine peritoneal macrophages infected with *Trypanosoma cruzi*—Concentration for drug: >12.0 μg/mL	Inactive [76]
			*In vitro*-Blood stream form trypomastigotes of *T. brucei rhodesiense*—IC_50_ for drug: 2.19 μg/mL	Active [76]
		EtOH (90%) ext. of leaves, root bark or stem bark	*In vitro*—Blood stream form trypomastigotes of *T. brucei rhodesiense*—Concentration for drugs: >25 μg/mL	Inactive [4]
		H_2_O ext. of leaves	*In vitro*-Blood stream form trypomastigotes of *T. brucei rhodesiense*—IC_50_ for drug: 10 μg/mL	Active [121]
	*C. quadrangulare* Kurz.	Acetone ext. of dried leaf	*In vitro*-Epimastigotes of *T. cruzi*-IC_50_ for drug: 6.25 μg/mL	Active [102]
		MeOH or H_2_O ext. of dried leaf	*In vitro*-Epimastigotes of *T. cruzi*—Concentration for drugs: 100.0 μg/mL	Inactive [102]
	*C. racemosum* P. Beauv	EtOH (90%) ext. of leaves or of root bark	*In vitro*-Blood stream form trypomastigotes of *T. brucei rhodesiense*—Concentration for drugs: >25 μg/mL	Inactive [4]
Larvicidal-Dengue fever				
	*C. aculeatum* Vent.	MeCl_2_, MeOH and H_2_O ext. of dried leaf or dried root bark	*In vitro*-Larvae of *Aedes aegypti*—Concentration for drugs: 500.0 μg/mL or 500.0 ppm	Inactive [122]
		MeOH and H_2_O ext. of dried stem	*In vitro*-Larvae of *A. aegypti*—Concentration for drugs: 500.0 ppm	Inactive [122]
	*C. collinum *Fresen.	Ether ext. of shoot bark	*In vitro*-Larvae of *A. aegypti*—Concentration for drug: 0.0125–0.200 mg/mL	Active [123]
*Antimicrobial activity*				
Antibacterial				
	*C. apiculatum Sond. ssp. apiculatum*	Hexane ext. of dried leaf	Microdilution assay (*Bacillus subtilis, Escherichia coli*, *Staphylococcus aureus* or *Klebisiella pneumoniae*)—Maximum concentration for drug: 12.5 mg/mL	Inactive [65]
		EtOH ext. of dried leaf	Microdilution assay (*B. subtilis* or *S. aureus* with MIC for drug: 0.049 mg/mL)	Active [65]
			Microdilution assay (*K. pneumonia *or *E. coli*)—Maximum concentration for drug: 12.5 mg/mL	Inactive [65]
		H_2_O ext. of dried leaf	Microdilution assay (*B. subtilis* or *S. aureus* with MIC for drug: 0.39 mg/mL )	Active [65]
			Microdilution assay (*K. pneumonia *or *E. coli*)—Maximum concentration for drug: 12.5 mg/mL	Inactive [65]
	*C. bracteatum *(Laws.) Engl. et Diels	EtOH (40%) or H_2_O ext. of dried stem	Agar diffusion method (*E. coli, Nisseria gonorrheae, S. aureus*, *Streptococcus* sp, *Salmonella typhimurium, B. subtilis, Bacteroides melaninogenicus*, *Clostridium tetani*, *Proteus vulgaris*, *Pseudomonas pyocyanea*, *Shgella dysenteriae *or *Yersinia enterocolita*)—Concentration for drugs: 0.33 g/mL	Inactive [124]
			Agar diffusion method (*K. pneumoniae *or *Bacteroides fragilis*)—Concentration for drugs: 0.33 g/mL with 5–9 mm diameter zone of inhibition	Active [124]
		EtOH (40%) ext. of dried stem	Agar diffusion method (*Corynebacterium diphtheriae*)—Concentration for drug: 0.33 g/mL	Inactive [124]
		H_2_O ext. of dried stem	Agar diffusion method (*C. diphtheriae*)—Concentration for drug: 0.33 g/mL with 5–9 mm diameter zone of inhibition	Active [124]
	*C. collinum* Fresen.	MeOH, EtOH or MeOH-H_2_O ext. of dried air parts	Agar diffusion method with diameters inhibition zones (*Pseudomonas aeruginosa)*—Concentraton for drugs: 1 and 5 mg/mL with inhibition of 9 mm	Active [57]
			Agar diffusion method with diameters inhibition zones (*E. coli, K. pneumoniae, Citrobacter freundii, S. aureus, Streptococcus pyogenes, Listeria monocytogenes *or *B. subtilis*)—Concentraton for drugs: 1 and 5 mg/mL	Inactive [57]
	*C. comosum *G. Don.	Hot H_2_O ext. of dried root bark	Agar diffusion method with diameters inhibition zones (*Mycobacterium phlei*, *Sarcina lutea* or *S. aureus*)—Concentration not cited.	Active [52]
	*C. erythrophyllum* (Burch.) Sond.	Acetone ext. of dried leaf	Microdilution assay—(*S. aureus*, *P. aeruginosa, Enteroccus faecalis *or *E*. *coli*)—IC_50_ for drug: 1.50, 1.50,1.50 or 0.8 mg/mL, respectively	Active [89]
		Acetone, EtOH (100%), CHCl_3_/MeOH/H_2_O (12:5:3), H_2_O, MeCl_2_ or MeOH ext. of dried leaf	Diluition and bioautographic TLC system assay (*S. aureus*)—Concentration for drugs: 0.1 g/mL	Active [125]
		CHCl_3_ or CCl_4_ ext. of freeze-dried leaf	Microdilution assays [(*S. aureus*, MIC for drugs: 0.1or 1.6 mg/mL, respectively), (*E. faecalis*, MIC for drugs: 0.2 or 1.6 mg/mL, respectively), (*E. coli*, MIC for drugs: 3.1 or 12.5 mg/mL, respectively) and (*P. aeruginosa*, MIC for drugs: 3.1 or 25.0 mg/mL, respectively)]	Active [88]
		H_2_O or MeOH/H_2_O (2:1) ext. of freeze-dried leaf	Microdilution assays [(*S. aureus*, MIC for drugs: 0.2 or 0.05 mg/mL, respectively), (*E. faecalis*, MIC for drugs: 0.4 mg/mL), (*E. coli*, MIC for drugs: 1.6 or 6.3 mg/mL, respectively), (*P. aeruginosa*, MIC for drugs: 6.3 or 12.5 mg/mL, respectively)]	Active [88]
		Butanol or Hexane ext. of freeze-dried leaf	Microdilution assays [(*S. aureus*, MIC for drugs: 0.4 or 50 mg/mL, respectively), (*E. faecalis*, MIC for drugs: 0.2 or 1.6 mg/mL, respectively), (*E. coli*, MIC for drugs: 25 or 0.8 mg/mL, respectively), (*P. aeruginosa* (MIC for drugs: 12.5 or 1.6 mg/mL, respectively)]	Active [88]
		CHCl_3_ fraction of leaves	Serial dilution microplate assay (*Micrococcus luteus, Shigella sonnei*, *Vibrio cholera* or *E. faecalis*—MIC for drug: 50, 25, 50 or 50 μg/mL, respectively)	Active [12]
	*C. glutinosum* Perrot. ex DC.	MeOH ext. of dried leaf	Agar diffusion method ( *S. lutea *and *E. coli *—Concentration for drug:15.0 and 10.0 mg/mL, respectively)	Active [53]
	*C. hartmannianum *Schweinf.	MeCl_2_, EtOAc or EtOH ext. of dried leaf	Microdilution assay (*B. subtilis*)—MIC for drugs: <0.1, 0.39 or 0.2 mg/mL, respectively	Active [106]
			Microdilution assay (*K. pneumonia*)—MIC for drugs: 0.2, 0.78 or 0.39 mg/mL, respectively	Active [106]
			Microdilution assay (*S. aureus*)—MIC for drugs: 1.56, 1.56 or 0.2 mg/mL, respectively	Active [106]
			Microdilution assay (*E. coli*)—MIC for drugs: 1.56, 1.56 or 0.39 mg/mL, respectively	Active [106]
		MeCl_2_, EtOAc or EtOH ext. of dried bark	Microdilution assay (*K. pneumonia*)—MIC for drugs: 0.39, 0.78 or 0.78 mg/mL, respectively	Active [106]
			Microdilution assay (*Staphylococcus aureus*)—MIC for all drugs: 3.13 mg/mL	Active [106]
			Microdilution assay (*E. coli*)—MIC for drugs: 3.13, 3.13 or 1.56 mg/mL, respectively	Active [106]
			Microdilution assay (*B. subtilis*)—MIC for drugs: 3.13, 0.39 or 1.56 mg/mL, respectively	Active [106]
			Microdilution assay (*K. pneumonia*)—MIC for drugs: 0.78, 0.78 or 0.2 mg/mL, respectively	Active [106]
			Microdilution assay (*B. subtilis*)—MIC for drugs: 0.1, 0.39 or 0.39 mg/mL, respectively	Active [106]
			Microdilution assay (*S. aureus*)—MIC for all drugs: 3.13, 3.13 or 0.2 mg/mL, respectively	Active [106]
			Microdilution assay (*E. coli*)—MIC for drugs: 3.13, 3.13 or 0.2 mg/mL, respectively	Active [106]
			Broth microdilution method (*Mycobacterium aurum* A+)—MIC for drugs: 0.78, 3.12 or 0.19 mg/mL, respectively	Active [126]
			Broth microdilution method (*M. aurum* A+)—MIC for drugs: 12.5, 25 or 1.56 mg/mL, respectively	Active [126]
		MeCl_2_ or EtOH ext. of dried root	Broth microdilution method (*M. aurum* A+)—MIC for drugs: 3.12 or 12.5 mg/mL, respectively	Active [126]
		EtOAc ext. of dried root	Broth microdilution method (*M. aurum* A+)—Concentration for drug: 25 mg/mL	Inactive [126]
	*C. imberbe* Wawra	MeCl_2_ ext. of dried leaves	Microplate serial dilution method (*S. aureus*)—Concentration for drug: 39 μg/mL	Active [127]
	*C. micranthum* G. Don	Hot H_2_O ext. of dried root	Agar diffusion method (*Mycobacterium phlei*)—Concentration not cited.	Inactive [52]
		MeOH, EtOH or MeOH-H_2_O ext. of dried air parts	Agar diffusion method with diameters inhibition zones (*P. aeruginosa*)—Concentration for drugs: 1 and 5 mg/mL with inhibition zone of 9 or 8 mm	Active [57]
			Agar diffusion method with diameters inhibition zones (*S. pyogenes, L. monocytogenes*)—Concentration for drugs: 1 mg/mL	Inactive [57]
			Agar diffusion method with diameters inhibition zones (*E. coli, K. pneumoniae, C. freundii *or *B. subtilis*)—Concentration for drugs: 1 and 5 mg/mL	Inactive [57]
			Agar diffusion method with diameters inhibition zones or microdilution assay (*S. aureus*)—Concentration for drugs: 1 and 5 mg/mL with inhibition zone of 10 mm, or MIC for drugs: 0.5 µg/mL	Active [57]
		EtOH (95%) ext. of dried twigs	Agar diffusion method with diameters inhibition zones (*B. subtilis* or *S. aureus*)—Concentration for drug: 50 mg/mL with inhibition zone > 15 mm or 5 mg/mL with inhibition zone > 15 mm	Active [49]
		EtOH (100%) ext. of dried leaf	Microplate serial dilution method [*Salmonella* sp, *Streptococcus* sp, *P. vulgaris*, *S. aureus*, *E. coli*, *P. aeruginosa *or *Klebsiella *sp—MIC for drug: 1.0 mg/mL]	Active [56]
		CHCl_3_ ext. of dried leaf	Microplate serial dilution method [*Salmonella* sp, *E. coli*, *P. aeruginosa*, *Klebsiella* sp—Concentration for drug: 1.0 mg/mL]	Inactive [56]
			Microplate serial dilution method [*Streptococcus* sp, *P. vulgaris* or *S. aureus*—MIC for drug: 1.0 mg/mL]	Active [56]
		H_2_O ext. of dried leaf	Microplate serial dilution method [*Salmonella* sp, *P. aeruginosa* or *S. aureus*—MIC for drug:1.0 mg/mL]	Active [56]
			Microplate serial dilution method [*E. coli*, *Klebsiella* sp, *Streptococcus* sp. or *P. vulgaris*—Concentration for drug: 1.0 mg/mL]	Inactive [56]
		MeOH ext. of dried leaf	Microplate serial dilution method (*S. lutea* or *E. coli*)—MIC for drug: 10.0 mg/mL	Active [53]
		Hot H_2_O ext. of dried root	Microplate serial dilution method [*S. lutea* or *S. aureus*]—Concentration not cited.	Active [52]
			Microplate serial dilution method (*C. diphtheria*)—MIC for drug: 5.0 mg/mL	Active [55]
		H_2_O ext. of dried root	Microplate serial dilution method (*Serratia marcescens* or *Salmonella typhosa* )-MIC for drug: 5.0 or 3.0 mg/mL, respectively	Active [55]
		Decoction of dried root	Microplate serial dilution method (*L. monocytogenes*, *E. faecalis*, *S. marcescens*, *S. typhosa* or *C. diphtheria*)—MIC for drug: 7.0, 7.0, 5.0, 3.0 or 3.0 mg/mL, respectively	Active [55]
		Decoction or H_2_O ext. of dried root	Microplate serial dilution method [*K. pneumonia* (MIC 5.0 mg/mL or 7.0 mg/mL, respectively); *S. aureus* ( MIC 1.0 mg/mL or 2.0 mg/mL, respectively)]	Active [55]
		H_2_O ext. of dried root	Microplate serial dilution method [*L. monocytogenes* ( MIC > 10.0 mg/mL), *E. faecalis* ( MIC > 10.0 mg/mL)	Inactive [55]
		Decoction or H_2_O ext. of dried root	Microplate serial dilution method [*M. luteus*, *P. aeruginosa*, *E. coli* or *B. subtilis*)—MIC for drug: 1.0, 5.0, 5.0, 5.0 or 5.0 mg/mL, respectively	Active [55]
		EtOH (100%) ext. of dried stembark	Microplate serial dilution method (*Salmonella* sp, *E. coli*, *P. vulgaris* or *Klebsiella *sp)—Concentration for drug: >1.0 mg/mL	Inactive [56]
			Microplate serial dilution method (*P. aeruginosa*, *S. aureus* or *Streptococcus* sp)—MIC for drug:1.0 mg/mL	Active [56]
		EtOH (95%) ext. of sun dried twig	Microplate serial dilution method (*B. subtilis* or *S. aureus*)—MIC for drug: 50.0 or 5.0 mg/mL, respectively)	Active [54]
	*C. molle* R.Br. ex G. Don.	Acetone and H_2_O ext. of dried bark	*In vitro*-Radiometric method (*M. tuberculosis*)—MIC for drugs: 1.0 mg/mL	Active [74]
		Acetone fraction of dried stem bark	Microdilution method (*M. tuberculosis* typus humanus)—Concentration for drug: 1.0–2 mg/mL	Inactive [128]
		MeOH ext. of dried bark	Microdilution method (*Streptococcus mutans* or *Actinomyces viscosus*)-MIC for drug:5.0 mg/mL	Active [129]
		Acetone ext. of dried leaf	Microdilution method (*S. aureus*)—MIC for drug: 0.07 mg/mL	Active [69]
		Acetone ext. of dried stembark	Agar diffusion method (*S. aureus*)—Concentration for drug: 1.0 mg/mL	Active [128]
		MeOH ext. of dried wood	Agar diffusion method (*S. mutans*)—Concentration for drug: 5.0 mg/disc	Inactive [129]
			Agar diffusion method (*A. viscosus*)—Concentration for drug: 5.0 mg/disc	Active [129]
		Acetone ext. of stem bark	Agar diffusion and micro broth dilution methods (*Helicobacter pylori*)—Concentration for drug: 100 mg/mL with inhibition zone of 10–38 mm, and MIC for drug: 0.08–2.50 mg/mL	Active [71]
		EtOH or MeOH ext. of stem bark	Agar diffusion method and micro broth dilution methods (*H. pylori*)—Concentration for drug: 100 mg/mL with inhibition zone of 7–35 or 7–32 mm	Active [71]
		AcOEt or H_2_O ext. of stem bark	Agar diffusion and micro broth dilution methods (*H. pylori*)—Concentration for drug: 100 mg/mL with inhibition zone of 0–21 or 0–20 mm	Active [71]
		EtOH ext. of stem bark	Agar dilution method (*Bacillus cereus* or *S. aureus*)—MIC for drug: 250 μg/mL	Active [70]
		MeOH ext. of dried root	Plate-hole diffusion and broth microdilution (*S. aureus*)—MIC for drug: 1 mg/mL	Active [130]
			Plate-hole diffusion and broth microdilution (*S. epidermidis*)—Concentration for drug: 1 mg/mL	Inactive [130]
		H_2_O ext. of dried root	Plate-hole diffusion and broth microdilution (*S. epidermidis* or *S. aureus*)—Concentration for drug: 1 mg/mL	Inactive [130]
		EtOH ext. of dried seed or stem	Agar plate with diameters inhibition zones—*S. aureus*—Concentration for drugs: 100 or 50 mg/mL with inhibition zone of 5 mm	Active [131]
		EtOH ext. of dried bark or leaf	Agar plate with diameters inhibition zones—*S. aureus*—Concentration for drugs: 3–100 mg/mL with inhibition zone of 20 mm	Active [131]
		EtOH ext. of dried leaf	Agar plate with diameters inhibition zones—*S. agalactiae*—Concentration for drug: 50 mg/mL with inhibition zone of 20 mm	Active [131]
	*C. paniculatum* Vent.	EtOH (80%) ext. of dried leaf	Microdilution method (*M. tuberculosis*)—Concentration for drug: 2 mg/mL	Inactive [128]
		Acid-EtOH ext. of dried leaf	Agar plate well-diffusion method (*S. aureus, Salmonella gallinarum*, *E. coli*, *P. vulgaris*, *P. aeruginosa*, *K. pneumonia*)—Concentration for drug: 0.20 mL/disc (1,000 µg/mL)	Active [132]
		H_2_O ext. of dried leaf	Agar plate well-diffusion method (*S. aureus*, *E. coli*, *P. vulgaris* or *K. pneumonia*)—Concentration for drug: 0.20 mL/disc (1,000 µg/mL)	Active [132]
			Agar plate well-diffusion method (*S. gallinarum* or *P. aeruginosa*)—Concentration for drug: 0.20 mL/disc (1,000 µg/mL)	Inactive [132]
		MeOH ext. of dried root	Plate-hole diffusion and broth microdilution—*S. epidermidis* (MIC for drug: 2.77 mg/mL) or *S. aureus* (MIC for drug: 1.85 mg/mL)	Active [130]
		H_2_O ext. of dried root	Plate-hole diffusion and broth microdilution—*S. epidermidis* or *S. aureus* (MIC for drug: 14.44 mg/mL)	Active [130]
	*C. quadrangulare *Kurz.	MeOH or H_2_O ext. of dried leaf	Agar plate well-diffusion method (*H. pylori*)—Concentration not cited	Active [100]
		EtOH (95%) ext. of dried seed or dried root	Agar plate well-diffusion method (several gram + organisms)—Concentration not cited	Active [99]
	*C. racemosum *P. Beauv.	EtOH (40%) or H_2_O ext. of dried petiole and leaves	Agar plate diffusion method (*E. coli*, *N. gonorrheae, Streptococcus* sp, *B. subtilis*, *P. vulgaris*, *P. pyocyanea, K. pneumoniae*, *B. fragilis, Y. enterocolita* or *S. typhimurium*)—Concentration for drugs: 0.33 g/mL	Inactive [124]
		EtOH (40%) ext. of dried petiole and leaves	Agar plate diffusion method (*S. aureus*)—Concentration for drugs: 0.33 g/mL with ≥ 20 mm diameter zone of inhibition	Active [124]
		EtOH (40%) ext. of dried leaf and stem	Agar plate diffusion method (*C. diphtheria*, *B. melaninogenicus* or *S. dysenteriae*)—Concentration for drugs: 0.33 g/mL with ≥ 20 mm diameter zone of inhibition	Active [124]
			Agar plate diffusion method (*C. tetani*)—Concentration for drugs: 0.33 g/mL with 10–19 mm diameter zone of inhibition	Active [124]
		H_2_O ext. of dried petiole and leaves	Agar plate diffusion method (*C. tetani*)—Concentration for drugs: 0.33 g/mL	Inactive [124]
	*C. raimbaultii *Heckel	EtOH/H_2_O (1:1) ext.	Agar plate diffusion method (*E. coli* or *S. aureus*)—Concentration not cited	Active [133]
			Agar plate diffusion method (*B. anthracis*)—Concentration not cited	Inactive [133]
	*C. zeyheri *Sond.	H_2_O ext. of fresh entire plant	Agar plate diffusion method (*N. gonorrhea*)—Concentration for drugs: 1.0 mg/mL	Inactive [134]
Antifungal				
	*C. aculeatum* Vent.	CHCl_3_, MeOH or H_2_O ext. of dried leaf or dried stem	Agar plate diffusion method (*Candida albicans*)—Concentration not cited	Active [135]
		MeOH, H_2_O or CHCl_3_ ext. of dried leaf or dried stem	Agar plate diffusion method (*Aspergillus niger*)—Concentration for drugs: 1 mg/mL	Active [135]
		MeCl_2_, MeOH or H_2_O ext. of dried leaf, dried root bark or dried stem	Agar plate diffusion method (*Cladosporium cucumerinum*)—Concentration for drugs: 100.0 μg/plate	Inactive [122]
	*C. acutifolium* Exell	Acetone, Hexane, MeCl_2_ or MeOH ext. of dried leaf	Microdilution assay [*C. albicans*—MIC for drugs: 0.16, 2.5, 2.5 or 0.04 mg/mL, respectively; *Criptococcus neoformans*—MIC for drugs: 0.04, 0.16, 0.16 or 0.08 mg/mL, respectively]	Active [73]
			Microdilution assay [*Aspergillus fumigates*—MIC for drugs: 0.08, 2.5, 0.16 or 0.16 mg/mL, respectively; *Sporothrix schenckii*—MIC for drugs: 0.04, 0.32, 0.32 or 0.08 mg/mL, respectively; *Microsporum canis*—MIC for all drugs: 0.02 mg/mL]	Active [73]
	*C. apiculatum *Sond. ssp. *apiculatum*	Acetone, Hexane, MeCl_2_ or MeOH ext. of dried leaf	Microdilution assay [*A. fumigates*—MIC for all drugs: 2.5 mg/mL; *M. canis*—MIC for all drugs: 0.02 mg/mL]	Active [73]
			Microdilution assay [*S. schenckii*—MIC for drugs: 0.02, 0.04, 0.02 or 0.02 mg/mL, respectively; *C. neoformans*—MIC for drugs: 0.08, 2.5, 0.08 or 0.08 mg/mL, respectively]	Active [73]
			Microdilution assay (*C. albicans*)—MIC for drugs: 0.32, 1.25, 0.32 or 0.32 mg/mL, respectively	Active [73]
	*C. albopuctatum*	Acetone, Hexane, MeCl_2 _or MeOH ext. of dried leaf	Microdilution assay (*C. albicans*)—MIC for drugs: 0.64, 2.5, 0.32or 0.32 mg/mL, respectively	Active [73]
			Microdilution assay (*C. neoformans*)—MIC for drugs: 0.08, 0.08, 0.16 or 0.16 mg/mL, respectively	Active [73]
			Microdilution assay (*A. fumigates*)—MIC for drugs: 0.08, 0.64, 0.16 or 0.32 mg/mL, respectively	Active [73]
			Microdilution assay (*S. schenckii*)—MIC for all drugs: 0.02 mg/mL	Active [73]
			Microdilution assay (*M. canis*)—MIC for drugs: 0.02, 0.02, 0.02 or 0.04 mg/mL, respectively	Active [73]
	*C. bracteosum* (Hochst.) Brandis ex Engl.	Acetone, Hexane, MeCl_2 _or MeOH ext. of dried leaf	Microdilution assay (*C. albicans*)—MIC for drugs: 1.25, 2.5, 2.5 or 1.25 mg/mL, respectively	Active [73]
			Microdilution assay (*C. neoformans*)—MIC for drugs: 0.16, 0.16, 0.32 or 0.32mg/mL, respectively	Active [73]
			Microdilution assay (*A. fumigates*)—MIC for all drugs: 2.5 mg/mL	Active [73]
			Microdilution assay (*S. schenckii*)—MIC for drugs: 0.16, 0.08, 0.16 or 0.16 mg/mL, respectively; *M. canis*—MIC for all drugs: 0.02 mg/mL	Active [73]
	*C. caffrum*	Acetone, Hexane, MeCl_2 _or MeOH ext. of dried leaf	Microdilution assay (*C. albicans*)—MIC for drugs: >2.5, 0.16, 0.64 or >2.5 mg/mL, respectively	Active [73]
			Microdilution assay (*C. neoformans*)—MIC for drugs: 0.32, 0.32, 0.16 or 0.32 mg/mL, respectively	Active [73]
			Microdilution assay (*A. fumigates*)—MIC for drugs: >2.5, 0.16 mg/mL, respectively	Active [73]
			Microdilution assay (*S. schenckii*)—MIC for drugs: 0.64, 0.64, 0.64 or 0.32 mg/mL, respectively	Active [73]
			Microdilution assay (*M. canis*)—MIC for drugs: 0.08, 0.32, 0.32 or 0.16 mg/mL, respectively	Active [73]
	*C. celastroides *Welw ex Laws subsp. *celastroides*	Acetone, Hexane, MeCl_2 _or MeOH ext. of dried leaf	Microdilution assay (*C. albicans*)—MIC for drugs: 0.16, 0.64, 0.32, 0.64 mg/mL, respectively	Active [73]
			Microdilution assay (*C. neoformans)*—MIC for drugs: 0.16, 0.16, 0.08 or 0.32 mg/mL, respectively	Active [73]
			Microdilution assay (*A. fumigates*)—MIC for drugs: 0.64, >2.5, 1.25 or 0.64 mg/mL, respectively	Active [73]
			Microdilution assay (*S. schenckii*)—MIC for drugs: 0.32, 0.32, 0.16 or 0.16 mg/mL, respectively	Active [73]
			Microdilution assay (*M. canis*)—MIC for drugs: 0.32, 0.64, 0.64 or 0.08 mg/mL, respectively	Active [73]
	*C. celastroides* Welw ex Laws subsp. *orientale*	Acetone, Hexane, MeCl_2 _or MeOH ext. of dried leaf	Microdilution assay (*C. albicans*)—MIC for drugs: 0.16, 0.32, 0.16 or 0.32 mg/mL, respectively	Active [73]
			Microdilution assay (*C. neoformans*)—MIC for drugs: 0.08, 0.32, 0.08 or 0.16 mg/mL, respectively	Active [73]
			Microdilution assay *(A. fumigates*)—MIC for drugs: 0.32, 2.5, 2.5 or 2.5 mg/mL, respectively	Active [73]
			Microdilution assay (*S. schenckii*)—MIC for drugs: 0.08, 0.16, 0.16 or 0.16 mg/mL, respectively	Active [73]
			Microdilution assay (*M. canis*)—MIC for drugs: 0.04, 0.32, 0.32 or 0.08 mg/mL, respectively	Active [73]
	*C. collinum *Fresen. subsp. *suluense *Okafor	MeOH ext. of dried root	Agar plate diffusion method-(*C. albicans* or *A. niger*)—Concentraton for drug: 1.0 mg/mL	Active [57]
		MeOH, EtOH or MeOH-H_2_O ext. of dried air parts	Agar plate with diameters inhibition zones (*C. albicans *or* A. niger*)—Concentraton for drugs: 5 mg/mL with inhibition zone of 10 or 14 mm, respectively	Active [57]
			Agar plate with diameters inhibition zones (*C. albicans or A. niger*)—Concentraton for drugs: 1 mg/mL	Inactive [57]
		Acetone, Hexane, MeCl_2 _or MeOH ext. of dried leaf	Microdilution assay (*C. albicans*)—MIC for drugs: 0.08, 2.5, 0.08 or 0.16 mg/mL, respectively	Active [73]
			Microdilution assay (*C. neoformans*)—MIC for drugs: 0.16, 2.5, 0.08 or 0.08 mg/mL, respectively	Active [73]
			Microdilution assay (*A. fumigates*)—MIC for all drugs: 2.5 mg/mL	Active [73]
			Microdilution assay (*S. schenckii*)—MIC for drugs: 0.16, 2.5, 0.16 or 0.32 mg/mL, respectively	Active [73]
			Microdilution assay (*M. canis*)—MIC for drugs: 0.64, 1.25, 0.64 or 0.32 mg/mL, respectively	Active [73]
	*C. collinum *Fresen. ssp *taborense*	Acetone, Hexane, MeCl_2 _or MeOH ext. of dried leaf	Microdilution assay (*C. albicans*)—MIC for all drugs: 0.64 mg/mL	Active [73]
			Microdilution assay (*C. neoformans*)—MIC for drugs: 0.08, 0.16, 0.32 or 0.32 mg/mL, respectively	Active [73]
			Microdilution assay (*A. fumigates*)—MIC for drugs: 0.64, 2.5, 2.5 or 2.5 mg/mL, respectively	Active [73]
			Microdilution assay (*S. schenckii*)—MIC for drugs: 0.64, 0.32, 0.32 or 0.64 mg/mL, respectively	Active [73]
			Microdilution assay (*M. canis*)—MIC for drugs: 0.32, 1.25, 0.64 or 0.16 mg/mL, respectively	Active [73]
	*C. comosum *G. Don.	Hot H_2_O ext. of dried root	Agar plate diffusion method (*Saccharomyces cerevisiae* or *A. niger*)—Concentration not cited	Inactive [52]
	*C. edwardsii* Exell	Acetone, Hexane, MeCl_2_ or MeOH ext. of dried leaf	Microdilution assay (*C. albicans*)—MIC for drugs: 0.32, 1.25, 1.25 or 0.64 mg/mL, respectively	Active [73]
			Microdilution assay (*C. neoformans*)—MIC for drugs: 0.04, 0.32, 0.32 or 0.16 mg/mL, respectively	Active [73]
			Microdilution assay (*A. fumigates)*- MIC for drugs: 2.5, 2.5, 2.5 or 2.5 mg/mL, respectively	Active [73]
			Microdilution assay (*S. schenckii*)—MIC for drugs: 0.04, 0.08, 0.08 or 0.04 mg/mL, respectively	Active [73]
			Microdilution assay (*M. canis*)—MIC for drugs: 0.04, 0.02, 0.04 or 0.04 mg/mL, respectively	Active [73]
	*C. erythrophyllum* (Burch.) Sond.	Acetone, Hexane, MeCl_2_ or MeOH ext. of dried leaf	Microdilution assay (*C. albicans*)—MIC for drugs: >2.5, 0.64, 0.64 or 2.5 mg/mL, respectively	Active [73]
			Microdilution assay (*C. neoformans*)—MIC for drugs: 2.5, 0.64, 0.32 or 0.64 mg/mL, respectively	Active [73]
			Microdilution assay (*A. fumigates*)—MIC for drugs: 2.5, >2.5, >2.5 or 2.5 mg/mL, respectively	Active [73]
			Microdilution assay (*S. schenckii*)—MIC for drugs: >2.5, 0.32, 0.32 or 1.25 mg/mL, respectively	Active [73]
			Microdilution assay (*M. canis*)—MIC for drugs: 0.02, 1.25, 0.32 or 0.16 mg/mL, respectively	Active [73]
	*C. glutinosum *Perrot. ex DC	EtOH/H_2_O (1:1) ext. of dried leaf	Microdilution assay (*C. albicans*, *Epidermophyton. floccosum*, *M. gypseum*, *Tricophyton mentagrophytes* or *Tricophyton rubrum*)—MIC for drug: >4.0, 4.0, 1.0, 1.0 or 1.0 mg/mL, respectively	Active [72]
	*C. hereroense *Schinz	Acetone, Hexane, MeCl_2_ or MeOH ext. of dried leaf	Microdilution assay (*C. albicans*)—MIC for drugs: 0.32, 0.32, 2.5 or 0.04, respectively	Active [73]
			Microdilution assay (*C. neoformans*)—MIC for drugs: 0.16, 0.08, 0.32 or 0.08 mg/mL, respectively	Active [73]
			Microdilution assay (*A. fumigates*)—MIC for drugs: 2.5, 2.5, 2.5 or 1.25 mg/mL, respectively	Active [73]
			Microdilution assay (*S. schenckii*)—MIC for drugs: 0.16, 0.16, 0.32 or 0.16 mg/mL, respectively	Active [73]
			Microdilution assay (*M. canis*)—MIC for drugs: 0.04, 0.02, 0.02 or 0.04 mg/mL, respectively	Active [73]
	*C. hispidum* Laws.	EtOH-H_2_O (1:1) ext. of dried leaf	Microdilution assay (*C. albicans*, *E. floccosum*, *M. gypseum*, *T. mentagrophytes* or *T. rubrum*)—MIC for drug: >4.0, >4.0, >4.0, 4.0 or 4.0 mg/mL, respectively	Active [72]
	*C. imberbe *Wawra	Acetone, Hexane, MeCl_2_ or MeOH ext. of dried leaf	Microdilution assay (*C. albicans*)—MIC for drugs: 2.5, 0.16, 0.16 or >2.5 mg/mL, respectively	Active [73]
			Microdilution assay (*C. neoformans*)—MIC for drugs: 0.16, 0.16, 0.32 or 0.32 mg/mL, respectively	Active [73]
			Microdilution assay (*A. fumigates*)—MIC for drugs: 2.5 mg/mL, respectively	Active [73]
			Microdilution assay (*S. schenckii*)—MIC for drugs: 2.5, >2.5, 0.32 or >2.5 mg/mL, respectively	Active [73]
			Microdilution assay (*M. canis*)—MIC for drugs: 0.32, 0.64, 0.16 or 0.32 mg/mL, respectively	Active [73]
	*C. kraussii *Hochst.	Acetone, Hexane, MeCl_2_ or MeOH ext. of dried leaf	Microdilution assay (*C. albicans*)—MIC for drugs: 2.5, 0.08, 0.32 or 1.25 mg/mL, respectively	Active [73]
			Microdilution assay (*C. neoformans*)—MIC for drugs: 0.64, 0.32, 0.16 or 0.32 mg/mL, respectively	Active [73]
			Microdilution assay (*A. fumigates*)—MIC for drugs: 0.64, 2.5, 2.5 or 0.16 mg/mL, respectively	Active [73]
			Microdilution assay (*S. schenckii*)—MIC for drugs: 0.64, 0.32, 0.32 or 0.64 mg/mL, respectively	Active [73]
			Microdilution assay (*M. canis*)—MIC for drugs: 0.32, 0.16, 0.64 or 0.04 mg/mL, respectively	Active [73]
	*C. micranthum *G. Don	MeOH, EtOH or MeOH-H_2_O ext. of dried air parts	Agar plate with diameters inhibition zones (*C. albicans*)—Concentraton for drugs: 5 mg/mL with inhibition zone of 11 mm	Active [57]
			Agar plate with diameters inhibition zones (*C. albicans*)—Concentraton for drugs: 1 mg/mL	Inactive [57]
			Agar plate with diameters inhibition zones (*A. niger*)—Concentraton for drugs: 1 or 5 mg/mL	Inactive [57]
		EtOH (95%) ext. of dried twigs	Agar plate with diameters inhibition zones (*A. niger*)—Concentration for drug: 50 or 5 mg/mL	Inactive [49]
		Hot H_2_O ext. of dried root	Agar plate diffusion method (*A. niger*)—Concentration not cited	Inactive [52]
		EtOH (95%) ext. of sun dried twig	Agar plate diffusion method (*A. niger*)—Concentration for drug: 50.0 mg/mL	Inactive [54]
		EtOH (100%) ext. of dried leaf	Agar plate diffusion method (*A. niger*)—Concentration for drug: 1.0 mg/mL	Inactive [57]
	*C. microphyllum* Klotzsch	Acetone, Hexane, MeCl_2_ or MeOH ext. of dried leaf	Microdilution assay (*C. albicans*)—MIC for all drugs: 2.5 mg/mL	Active [73]
			Microdilution assay (*C. neoformans*)—MIC for drugs: 0.16, 0.64, 0.08 or 0.16 mg/mL, respectively	Active [73]
			Microdilution assay ( *A. fumigates*)—MIC for all drugs: 2.5 mg/mL	Active [73]
			Microdilution assay (*S. schenckii*)—MIC for drugs: 0.64, 0.64, 0.32 or 0.32 mg/mL, respectively	Active [73]
	*C. moggi* Exell	Acetone, Hexane, MeCl_2_ or MeOH ext. of dried leaf	Microdilution assay (*C. albicans*)—MIC for drugs: 0.64, 1.25, 1.25 or 0.02 mg/mL, respectively	Active [73]
			Microdilution assay (*C. neoformans*)—MIC for drugs: 0.08, 0.32, 0.32 or 0.04 mg/mL, respectively	Active [73]
			Microdilution assay (*A. fumigates*)—MIC for all drugs: 2.5 mg/mL	Active [73]
			Microdilution assay (*S. schenckii*)—MIC for drugs: 0.02, 0.16, 0.08 or 0.02 mg/mL, respectively	Active [73]
			Microdilution assay (*M. canis*)—MIC for drugs: 0.04, 0.08, 0.04 or 0.02 mg/mL, respectively	Active [73]
	*C. molle* R.Br. ex G. Don.	MeOH ext. of dried bark	Microdilution assay (*C. albicans*)—MIC for drug: 5.0 mg/mL	Active [129]
		MeOH ext. of dried wood	Agar plate diffusom method (*C. albicans*)—Concentration for drug: 5.0 mg/disc	Inactive [129]
		EtOH/H_2_O (1:1) ext. of dried leaf	Microdilution assay (*C. albicans*, *E. floccosum*, *M. gypseum*, *T. mentagrophytes* or *T. rubrum*)—MIC for drug: > 4.0, 0.5, 0.25, 0.25 or 0.5 mg/mL, respectively	Active [72]
		MeOH ext. of dried root	Macro-broth tube dilution method (*C. albicans*)—MIC for drug: 1 mg/mL	Active [136]
		H_2_O ext. of dried root	Macro-broth tube dilution method (*C. albicans*)—MIC for drug: 6.50 mg/mL	Active [136]
		Acetone, Hexane, MeCl_2_ or MeOH ext. of dried leaf	Microdilution assay (*C. albicans*)—MIC for drugs: 0.04, 1.25, 0.32 or 0.32 mg/mL, respectively	Active [73]
			Microdilution assay (*C. neoformans*)—MIC for drugs: 0.04, 1.25, 0.16 or 0.08 mg/mL, respectively	Active [73]
			Microdilution assay (*A. fumigates*)—MIC for drugs: 1.25, 2.5, 2.5 or 2.5 mg/mL, respectively	Active [73]
			Microdilution assay (*S. schenckii*)—MIC for drugs: 0.08, 0.32, 0.32 or 0.08 mg/mL, respectively	Active [73]
			Microdilution assay (*M. canis*)—MIC for drugs: 0.02, 0.02, 0.04 or 0.02 mg/mL, respectively	Active [73]
	*C. mossambicense *(Klotzsch) Engl.	Acetone, Hexane, MeCl_2_ or MeOH ext. of dried leaf	Microdilution assay (*C. albicans*)—MIC for drugs: 1.25, 2.5, 2.5 or 1.25 mg/mL, respectively	Active [73]
			Microdilution assay (*C. neoformans*)—MIC for drugs: 1.25, 1.25, 0.64 or 0.64 mg/mL, respectively	Active [73]
			Microdilution assay (*A. fumigates*)—MIC for all drugs: 2.5 mg/mL	Active [73]
			Microdilution assay (*S. schenckii*)—MIC for drugs: 0.64, 0.16, 0.16 or 0.16 mg/mL, respectively	Active [73]
			Microdilution assay (*M. canis*)—MIC for drugs: 0.08, 0.04, 0.02 or 0.32 mg/mL, respectively	Active [73]
	*C. nelsonii *Dümmer	Acetone, Hexane, MeCl_2_ or MeOH ext. of dried leaf	Microdilution assay (*C. albicans*)—MIC for drugs: 0.04, 0.16, 0.32 or 0.16 mg/mL, respectively	Active [73]
			Microdilution assay (*C. neoformans*)—MIC for drugs: 0.16, 0.32, 0.32 or 0.16 mg/mL, respectively	Active [73]
			Microdilution assay (*A. fumigates*)—MIC for drugs: 0.64, 2.5, 0.64 or 0.64 mg/mL, respectively	Active [73]
			Microdilution assay (*S. schenckii* )—MIC for drugs: 0.08, 0.32, 0.16 or 0.16 mg/mL, respectively	Active [73]
			Microdilution assay (*M. canis*)—MIC for all drugs: 0.02 mg/mL	Active [73]
	*C. nigricans* Lepr.	EtOH/H_2_O (1:1) ext. of dried leaf	Microdilution assay (*C. albicans*, *E. floccosum*, *M. gypseum*, *T. mentagrophytes* or *T. rubrum*)—MIC for drug: >4.0, 1.0, 1.0, 1.0 or 1.0 mg/mL, respectively	Active [72]
		EtOH/H_2_O (1:1) ext. of dried entire root	Microdilution assay (*C. albicans*, *E. floccosum*, *M. gypseum*, *T. mentagrophytes* or *T. rubrum*)—MIC for drug: >4.0, 0.25, 0.5, 0.25 or 0.5 mg/mL, respectively	Active [72]
	*C. padoides *Engl. & Diels	Acetone, Hexane, MeCl_2_ or MeOH ext. of dried leaf	Microdilution assay (*C. albicans*)—MIC for drugs: 0.16, 0.32, 0.32 or >2.5 mg/mL, respectively	Active [73]
			Microdilution assay (*C. neoformans*)—MIC for drugs: 0.32, 0.64, 0.32 or 0.32 mg/mL, respectively	Active [73]
			Microdilution assay (*A. fumigates)*—MIC for drugs: 0.32, 2.5, 2.5 or 0.32 mg/mL, respectively	Active [73]
			Microdilution assay (*S. schenckii*)—MIC for drugs: 0.32, >2.5, >2.5 or 0.64 mg/mL, respectively	Active [73]
			Microdilution assay (*M. canis*)—MIC for drugs: 0.08, 0.64, 0.16 or 0.08 mg/mL, respectively	Active [73]
	*C. paniculatum* Vent.	Acid-EtOH or H_2_O ext. of dried leaf	Agar plate diffusion method (*C. albicans*)—Concentration for drug: 0.20 mL/disc (1,000 µg/mL)	Active [132]
		MeOH or H_2_O ext. of dried root	Macro-broth tube dilution method (*C. albicans*)—MIC for drugs: 5.55 or 14.44 mg/mL, respectively	Active [136]
		Acetone, Hexane, MeCl_2_ or MeOH ext. of dried leaf	Microdilution assay (*C. albicans*)—MIC for all drugs: 2.5 mg/mL	Active [73]
			Microdilution assay (*C. neoformans*)—MIC for drugs: 0.32, 1.25, 0.16 or 0.16 mg/mL, respectively	Active [73]
			Microdilution assay (*A. fumigates*)—MIC for all drugs: 2.5 mg/mL	Active [73]
			Microdilution assay (*S. schenckii*)—MIC for drugs: 0.32, 0.32, 0.04 or 0.04 mg/mL, respectively	Active [73]
			Microdilution assay (*M. canis*)—MIC for drugs: 0.02, 0.02, 0.02 or 0.08 mg/mL, respectively	Active [73]
	*C. petrophilum *Retief	Acetone, Hexane, MeCl_2_ or MeOH ext. of dried leaf	Microdilution assay (*C. albicans*)—MIC for drugs: 0.04, 2.5, 2.5 or 0.04 mg/mL, respectively	Active [73]
			Microdilution assay (*C. neoformans*)—MIC for drugs: 0.02, 0.32, 2.5 or 0.02 mg/mL, respectively	Active [73]
			Microdilution assay (*A. fumigates*)—MIC for all drugs: 2.5 mg/mL	Active [73]
			Microdilution assay (*S. schenckii*)—MIC for drugs: 0.08, 0.32, 0.32 or 0.04 mg/mL, respectively	Active [73]
			Microdilution assay (*M. canis*)—MIC for all drugs: 0.02, 0.04, 0.04 or 0.02 mg/mL, respectively	Active [73]
	*C. woodii* Dümmer	Acetone, Hexane, MeCl_2_ or MeOH ext. of dried leaf	Microdilution assay (*C. albicans*)—MIC for drugs: 0.16, 0.08, 0.16 or 0.32 mg/mL, respectively	Active [73]
			Microdilution assay (*C. neoformans*)—MIC for drugs: 0.32, 0.16, 0.16 or 2.5 mg/mL, respectively	Active [73]
			Microdilution assay (*A. fumigates*)—MIC for drugs: 1.25, 2.5, 1.25 or 2.5 mg/mL, respectively	Active [73]
			Microdilution assay (*S. schenckii*)—MIC for drugs: 0.32, 0.32, 0.32 or 1.25 mg/mL, respectively	Active [73]
			Microdilution assay (*M. canis*)—MIC for all drugs: 0.32 mg/mL	Active [73]
	*C. zeyheri *Sond.	MeOH ext. of dried entire plant	Agar plate diffusion method (*C. albicans *or *T. mentagrophytes*)—Concentration for drug: 0.03 mg/mL	Active [137]
		Acetone, Hexane, MeCl_2_ or MeOH ext. of dried leaf	Microdilution assay (*C. albicans*)—MIC for drugs: 0.16, 2.5, 1.25 or 0.16 mg/mL, respectively	Active [73]
			Microdilution assay (*C. neoformans*)—MIC for all drugs: 0.32 mg/mL	Active [73]
			Microdilution assay (*A. fumigates*)—MIC for all drugs: 2.5 mg/mL	Active [73]
			Microdilution assay (*S. schenckii*)—MIC for drugs: 0.02, 0.08, 0.04 or 0.08 mg/mL, respectively	Active [73]
			Microdilution assay (*M. canis*)—MIC for drugs: 0.02, 0.02, 0.02 or 0.04 mg/mL, respectively	Active [73]
*Hypoglycemic activity*				
	*C. decandrum* Roxb. (DC)	EtOH (70%) ext. of dried leaf	Streptozotocin-induced diabetic in rat—Dose for drug: 0.75 g/kg (*p.o.*)	Active [138]
	*C. micranthum *G. Don	H_2_O ext. of leaves	Induction of *Diabetes mellitus* Type 1 and 2 by alloxan in rats—Doses for drug: 100, 200 or 400 mg/kg (*p.o.*)	Active [63]
*Antiinflammatory activity*				
	*C. collinum *Fresen.	H_2_O ext. of dried stem bark	12-O-tetradecanoylphorbol-13-acetate (TPA)-induced ear inflammation in mice—Dose for drug: 0.5 mg/ear	Active [139]
			Carrageenan-induced pedal edema in mice—Dose for drug: 100.0 mg/kg (*p.o.*)	Active [139]
	*C. dolichopetalum* Gils ex Engl.	MeOH ext. of dried root	Carrageenan-induced paw edema in mice—Doses for drug: 200, 400 or 600.0 mg/kg (*p.o.*)	Active [96]
		CHCl_3_ ext. of dried root	Croton oil-induced ear edema in mice—Doses for drug: 0.25, 0.5 or 1.0 mg/ear	Active [96]
	*C. apiculatum* Sond. subsp. *apiculatum*	H_2_O, Acetone or AcOEt ext. of dried leaf	*In vitro*-Cyclooxygenase-1 (COX-1) inhibition by radioactivity bioassay—Concentration not cited	Active [112]
	*C*. *bracteosum *(Hochst.) Brandis ex Engl.	H_2_O, Acetone or AcOEt ext. of dried leaf	*In vitro*-Cyclooxygenase-1 (COX-1) inhibition by radioactivity bioassay—Concentration not cited	Active [112]
	*C*. *celastroides *Welw ex Laws subsp. *celastroides*	H_2_O, Acetone or AcOEt ext. of dried leaf	*In vitro*-Cyclooxygenase-1 (COX-1) inhibition by radioactivity bioassay—Concentration not cited	Active [112]
	*C*. *collinum *Fresen. subsp. *suluense *(Engl. & Diels) Okafor	H_2_O, Acetone or AcOEt ext. of dried leaf	*In vitro*-Cyclooxygenase-1 (COX-1) inhibition by radioactivity bioassay—Concentration not cited	Active [112]
	*C. duarteanum *Cambess.	EtOH ext. of dried leaf	Carrageenan or arachidonic acid-induced hind paw edema in mice—Doses for drug: 200 or 400 mg/kg (*i.p.*)	Active [140]
	*C*. *edwardsii *Exell	H_2_O, Acetone or AcOEt ext. of dried leaf	*In vitro*-Cyclooxygenase-1 (COX-1) inhibition by radioactivity bioassay—Concentration not cited	Active [112]
	*C*. *erythrophyllum* (Burch.) Sond.	H_2_O, Acetone or AcOEt ext. of dried leaf	*In vitro*-Cyclooxygenase-1 (COX-1) inhibition by radioactivity bioassay—Concentration not cited	Active [112]
	*C. hartmannianum *Schweinf.	MeCl_2_ or EtOH ext. of dried leaf	*In vitro*-Cyclooxygenase-1 (COX-1) inhibition by radioactivity bioassay—Concentration for all drugs: 250 μg/mL	Active [126]
		AcOEt ext. of dried leaf	*In vitro*-Cyclooxygenase-1 (COX-1) inhibition by radioactivity bioassay—Concentration for drug: 250 μg/mL	Inactive [126]
		MeCl_2_ or AcOEt ext. of dried bark	*In vitro*-Cyclooxygenase-1 (COX-1) inhibition by radioactivity bioassay—Concentration for all drugs: 250 μg/mL	Inactive [126]
		EtOH ext. of dried bark	*In vitro*-Cyclooxygenase-1 (COX-1) inhibition by radioactivity bioassay—Concentration for drug: 250 μg/mL	Active [126]
		MeCl_2_, AcOEt or EtOH ext. of dried root	*In vitro*-Cyclooxygenase-1 (COX-1) inhibition by radioactivity bioassay—Concentration for all drugs: 250 μg/mL	Inactive [126]
	*C*. *hereroense *Schinz	H_2_O, Acetone or AcOEt ext. of dried leaf	*In vitro*-Cyclooxygenase-1 (COX-1) inhibition by radioactivity bioassay—Concentration not cited	Active [112]
	*C*. *imberbe *Wawra	H_2_O, Acetone or AcOEt ext. of dried leaf	*In vitro*-Cyclooxygenase-1 (COX-1) inhibition by radioactivity bioassay—Concentration not cited	Active [112]
	*C*. *kraussii *Hochst.	H_2_O, Acetone or AcOEt ext. of dried leaf	*In vitro*-Cyclooxygenase-1 (COX-1) inhibition by radioactivity bioassay—Concentration not cited	Active [112]
	*C*. *microphyllum* Klotzsch	H_2_O, Acetone or AcOEt ext. of dried leaf	*In vitro*-Cyclooxygenase-1 (COX-1) inhibition by radioactivity bioassay—Concentration not cited	Active [112]
	*C*. *mkuzense *Carr & Retief	H_2_O, Acetone or AcOEt ext. of dried leaf	*In vitro*-Cyclooxygenase-1 (COX-1) inhibition by radioactivity bioassay—Concentration not cited	Active [112]
	*C*. *moggii *Exell	H_2_O ext. of dried leaf	*In vitro*-Cyclooxygenase-1 (COX-1) inhibition by radioactivity bioassay—Concentration not cited	Inactive [112]
		Acetone or AcOEt ext. of dried leaf	*In vitro*-Cyclooxygenase-1 (COX-1) inhibition by radioactivity bioassay—Concentration not cited	Active [112]
	*C*. *molle *R. Br. Ex G. Don	H_2_O, Acetone or AcOEt ext. of dried leaf	*In vitro*-Cyclooxygenase-1 (COX-1) inhibition by radioactivity bioassay—Concentration not cited	Active [112]
	*C*. *mossambicense *(Klotzsch) Engl.	H_2_O, Acetone or AcOEt ext. of dried leaf	*In vitro*-Cyclooxygenase-1 (COX-1) inhibition by radioactivity bioassay—Concentration not cited	Active [112]
	*C*. *nelsonii *Dümmer	H_2_O, Acetone or AcOEt ext. of dried leaf	*In vitro*-Cyclooxygenase-1 (COX-1) inhibition by radioactivity bioassay—Concentration not cited	Active [112]
	*C*. *padoides *Engl. & Diels	H_2_O, Acetone or AcOEt ext. of dried leaf	*In vitro*-Cyclooxygenase-1 (COX-1) inhibition by radioactivity bioassay—Concentration not cited	Active [112]
	*C*. *paniculatum *Vent.	H_2_O, Acetone or AcOEt ext. of dried leaf	*In vitro*-Cyclooxygenase-1 (COX-1) inhibition by radioactivity bioassay—Concentration not cited	Active [112]
	*C*. *petrophilum *Retief	H_2_O, Acetone or AcOEt ext. of dried leaf	*In vitro*-Cyclooxygenase-1 (COX-1) inhibition by radioactivity bioassay—Concentration not cited	Active [112]
	*C*. *woodii *Dümmer	H_2_O ext. of dried leaf	*In vitro*-Cyclooxygenase-1 (COX-1) inhibition by radioactivity bioassay—Concentration not cited	Inactive [112]
		Acetone or AcOEt ext. of dried leaf	*In vitro*-Cyclooxygenase-1 (COX-1) inhibition by radioactivity bioassay—Concentration not cited	Active [112]
	*C. hartmannianum *Schweinf.	MeCl_2_, EtOAc or EtOH ext. of dried leaf	*In vitro*-Cyclooxygenase-2 (COX-2) inhibition by radioactivity bioassay—Concentration for all drugs: 250 μg/mL	Active [126]
		EtOAc or EtOH ext. of dried bark	*In vitro*-Cyclooxygenase-2 (COX-2) inhibition by radioactivity bioassay—Concentration for all drugs: 250 μg/mL	Inactive [126]
		MeCl_2_, EtOAc or EtOH ext. of dried root	*In vitro*-Cyclooxygenase-2 (COX-2) inhibition by radioactivity bioassay—Concentration for all drugs: 250 μg/mL	Inactive [126]
	*C. micranthum* G. Don.	Hot H_2_O of dried aerial parts	Radioactivity assays of PGs isolated of stomach in rat—Concentration for drug: 100.0 µL/mL	Inactive [141]
		H_2_O ext. of dried leaf	Carrageenan-induced paw oedema or Cotton pellet granuloma formation in rats—Doses for drug: 50 or 100 mg/kg (*p.o.*)	Active [142]
			Acetic acid-induced vascular permeability in mice—Doses for drug: 50 or 100 mg/kg (*p.o.*)	Active [142]
*Antinociceptive activity*				
	*C. duarteanum* Cambess.	EtOH ext. of dried leaf	Acid-induced writhing, formalin, and hot-plate nociception tests in mice—Doses for drug: 100, 200, or 400 mg/kg (*i.p.*)	Active [140]
	*C. leprosum *Mart.	EtOH ext. of dried flowers	Formalin induced nociception in mice—Doses for drug: 100 and 300 mg/kg (*p.o*)	Active [7]
			Abdominal contortion by acetic acid in mice—Doses for drug: 30, 100, 300, 1.000 mg/kg (*p.o.*)	Active [7]
			Capsaicin-induced nociception in mice—Doses for drug: 30, 100, 300, 1.000 mg/kg (*p.o.*)	Active [7]
			Glutamate induced nociception in mice—Doses for drug: 10, 30, 100, 300 mg/kg (*p.o.*)	Active [7]
			Hot plate test in mice—Doses for drug: 10, 30, 100, 300 mg/kg (*p.o.*)	Active [7]
		EtOH (70%) ext. of dried stem bark	Tail immersion test and Formalin-induced pain in mice—Doses for drug: 25.0 mg/kg (*i.p.*) or 500.0 mg/kg (*p.o.*)	Active [143]
*Antioxidant activity*				
	*C. decandrum* Roxb. (DC)	EtOH (70%) ext. of dried leaf	Thiobarbituric acid-reactive substance or ferrous ion oxidation xylenol orange in rats—Dose for drug: 0.75 g/kg (*p.o.*)	Active [138]
	*C. duarteanum *Cambess.	EtOH ext. of dried leaf	Thiobarbituric acid-reactive substance, hydroxyl radical-scavenging, or scavenging activity of nitric oxide assays.	Active [140]
*Anti-tumour activity*				
	*C. caffrum* (Eckl. and Zeyh.) Kuntze	CHCl_3_, CCl_4_ or CH_2_Cl_2_ fractions of dried fruit, leaf, stem or twig	*In vitro*-Cell culture (immature astrocytoma 224c glioma cell)—Concentration for drugs: 1.0–100 μg/mL	Active [144]
		CCl_4_ or CH_2_Cl_2_ fraction of dried fruit, leaf, stem or twig	*In vitro*-P388 lymphocytic leukemia cell growth inhibition (ED_50_ for drugs: 1.5 or 0.23 µg/mL, respectively)	Active [144]
			Murine P-388 lymphocytic leukemia cell growth inhibition—Doses for drugs (*i.p.*): 100 or 50 mg/kg, respectively	Active [144]
		MeCl_2_ ext. of dried root bark	Murine P-388 lymphocytic leukemia cell growth inhibition—Dose not cited (*i.p.*).	Active [145]
	*C. collinum* Fresen.	MeOH, EtOH or MeOH-H_2_O ext. of dried air parts	*In vitro*-Cell culture (Squamous carcinoma KB, Melanoma SK—MEL28, lung carcinoma A549, or mamma carcinoma MDA—MB231)-IC_50_ for all drugs: 20.0 μg/mL	Active [57]
*Antitussive activity*				
	*C. glutinosum *Perrot. ex DC	H_2_O ext. of dried leaf	Guinea pig—Dose for drug: 1.0 mg/kg (*p.o.*)	Active [146]
*Antiviral activity*				
	*C. glutinosum *Perrot. ex DC	Decoction of leaf	*In vitro*-Cell culture (hepatitis B virus antigen HBsAg-IC_50_ for drug:100.0–500 ng/mL	Active [147]
	*C. grandiflorum* G. Don	EtOH (80%) ext	*In vitro*-Cell culture (plaque-inhibition in cells infected with virus-*Adenovirus*)—Concentration not cited	Inactive [148]
		EtOH (80%) ext	*In vitro*-Cell culture (plaque-inhibition in cells infected with virus *Herpes* type 1)—Concentration for drug:	Inactive [148]
			In vitro-Cell culture (plaque-inhibition in cells infected with virus measles)—Concentration not cited	Inactive [148]
			In vitro-Cell culture (plaque-inhibition in cells infected with virus *Poliovirus* I)—Concentration not cited	Inactive [148]
		EtOH (80%) ext	*In vitro*-Cell culture (plaque-inhibition in cells infected with virus *Coxsackie* B2)—Concentration for drug:	Inactive [148]
			*In vitro*-Cell culture (plaque-inhibition in cells infected with virus *Semlicki forest*)—Concentration not cited	Inactive [148]
	*C. micranthum *G. Don.	MeOH ext. of dried leaf	*In vitro*-Cell culture: African green monkey cells infected with virus *Herpes simplex* 1 or *H. simplex* 2—Concentration for drug: 7.5 μg/mL	Active [58]
	*C. paniculatum *Vent.	MeOH ext. of dried leaf	*In vitro-*Cell culture: MT-4 cells infected with virus human immunodeficiency type 1 (HIV 1)—IC_50_ for drug: 5.2 μg/mL	Active [149]
			*In vitro-*Cell culture: MT-4 cells infected with virus HIV 2 (rod)—Concentration for drug: >24.6 μg/mL	Inactive [149]
		EtOH (80%) ext. of dried leaf	*In vitro-*Cell culture: MT-4 cells infected with virus HIV 1 or HIV 2 (ROD)—Concentration for drug: >23.5 μg/mL	Inactive [149]
		Pet ether ext. of dried leaf	*In vitro-*Cell culture: MT-4 cells infected with virus HIV 1 or HIV 2 (ROD)—Concentration for drug: >118 μg/mL	Inactive [149]
		MeCl_2_ ext. of dried leaf	*In vitro-*Cell culture: MT-4 cells infected with virus HIV 1 or HIV 2 (ROD)—Concentration for drug: >44.7 μg/mL	Inactive [149]
		Acetone ext. of dried leaf	*In vitro-*Cell culture: MT-4 cells infected with virus HIV 1 or HIV 2 (ROD)-IC_50_ for drug: 15.0 or 3.0 μg/mL, respectively	Active [149]
	*C. quadrangulare *Kurz.	EtOH (95%) or H_2_O ext. of dried leaf	*In vitro-*HIV 1 integrase inhibition by cell culture with virus HIV 1)-IC_50_ for drugs: 2.5 or 2.9 μg/mL, respectively	Active [103]
	*C. molle *R. Br. Ex G. Don	H_2_O or MeOH ext. of roots	*In vitro-*RNA-dependent-DNA polymerase (RDDP) activity of HIV1 reverse transcriptase-IC_50_ for drugs: 37or 9.7 μg/mL, respectively	Active [80]
*Immunostimulant activity*				
	*C. micranthum *G. Don	Suspension of powder leaf	Rate of clearance of colloidal carbon by mice—Dose for drug: 100.0 mg/kg (*i.v.*)	Active [62]
*Cardiovascular activity*				
	*C. hypopilinum* Diels	MeOH ext. of seed	Depressant cardiac in rabbit - Dose not cited	Active [150]
	*C. nigricans *Lepr.	MeOH ext. of seed	Rabbit-heart- Dose not cited	Active [150]
	*C. sokodense* Engl.	MeOH ext. of seed	Rabbit-heart- Dose not cited	Active [150]
	*C. verticillatum* Engl. & Diels	MeOH ext. of seed	Rabbit-heart- Dose not cited	Active [150]
	*C. racemosum* P. Beauv	Hot H_2_O ext. of dried leaf	Blood pressure blocked by DHE in cat—Dose for drug: 0.5 mL/kg (*i.v.*)	Inactive [151]
	*C. hypopilium* Diels	MeOH ext	Hypotensive in cat—Dose for drug: 250.0 mg/kg (*i.v.*)	Active [150]
	*C. nigricans *Lepr.	MeOH ext. of seed	Hypotensive in cat—Dose for drug: 250.0 mg/kg (*i.v.*)	Active [150]
	*C. ovalifolium var.cooperi*	EtOH/H_2_O (1:1) ext. of aerial parts	Cat—Dose for drug: 50.0 mg/kg (*i.v.*)	Active [152]
	*C. sokodense* Engl.	MeOH ext. of seed	Hypotensive in cat—Dose for drug: 250.0 mg/kg (*i.v.*)	Active [150]
	*C. verticillatum* Engl. & Diels	MeOH ext. of seed	Hypotensive in cat—Dose for drug: 250.0 mg/kg (*i.v.*)	Active [150]
*CNS activity*				
	*C. hypopilium* Diels	MeOH ext. of seed	Depressant CSN im mice—Dose for drug:0.5 mg/kg (*i.p.*)	Active [150]
	*C. nigricans *Lepr.	MeOH ext. of seed	Depressant CSN im mice—Dose for drug: 0.5 mg/kg (*i.p.*)	Active [150]
	*C. paniculatum *Vent.	MeOH ext. of seed	Stimulate CSN im mice—Dose for drug: 0.5 mg/kg (*i.p.*)	Active [150]
	*C. sokodense* Engl.	MeOH ext. of seed	Depressant CSN im mice—Dose for drug: 0.5 mg/kg (*i.p.*)	Active [150]
	*C. verticillatum* Engl. & Diels	MeOH ext. of seed	Depressant CSN im mice—Dose for drug: 0.5 mg/kg (*i.p.*)	Active [150]
*Toxicity studies*				
Mutagenicity				
	*C. erythrophyllum* (Burch.) Sond.	H_2_O ext. of dried root	*In vitro*-Agar plate with *S. typhimurium* TA97a and TA98-Concent. for drug: 100.0–20.0 μg/disc	Inactive [90]
			*In vitro*-Agar plate with *S. typhimurium *TA100 and TA102—Concentration for drug: 40.0, 70.0, 80.0, 90.0, 100.0 μg/disc	Active [90]
			*In vitro*-Spermatocytes drosophila sex-linked recessive lethal concentration 50% (LD_50_)—Dose for drug: 1.0 mg/mL	Active [86]
Cytotoxicity				
	*C. apiculatum* Sond. subsp *apiculatum*	MeOH ext. of dried leaf	*In vitro*-Cell culture (T24 bladder or MCF7 breast cancer)—Concentration of drug: 25 µg/mL	Active [15]
		MeOH ext. of dried root	*In vitro*-Cell culture (T24 bladder, HeLa cervical or MCF7 breast cancer)—Concentration of drug: 25 µg/mL	Active [15]
	*C. aculeatum* Vent.	MeCl_2_, MeOH, H_2_O ext. of dried leaf	*In vitro*-Cell culture Concentration for drugs: 500.0 μg/mL or 500.0 ppm	Inactive [122]
		H_2_O ext. of dried root	*In vitro*-Cell culture (SW480 colon cancer cells)—Concentration for drug: 500.0 ppm	Inactive [122]
		MeCl_2_ ext. of dried root	*In vitro*-Cell culture (CO115 colon cancer cells)—Concentration for drug: 500.0 μg/mL	Inactive [122]
		MeOH or H_2_O ext. of dried root	*In vitro*-Cell culture (CO115 colon cancer cells)—Concentration for drugs: 500.0 ppm	Inactive [122]
		MeCl_2_ ext. of dried stem	*In vitro*-Cell culture (SW480 colon cancer cells or CO115 colon cancer cells)—Concentration for drug: 500.0 μg/mL	Inactive [122]
		MeOH or H_2_O ext. of dried stem	*In vitro*-Cell culture (SW480 colon cancer cells or CO115 colon cancer cells)—Concentration for drugs: 500.0 ppm	Inactive [122]
	*C. collinum *Fresen.	MeOH ext. of dried leaf	Cell culture (T24 bladder or MCF7 breast cancer)—Concentration for drug: 25 µg/mL	Active [15]
		MeOH ext. of dried root	Cell culture (T24 bladder, HELA cervical or MCF7 breast cancer)—Concentration for drug: 25 µg/mL	Active [15]
	*C. comosum *G. Don.	MeOH, MeOH/H_2_O (50:50) or MeCl_2 _ext. of dried leaves	*In vitro*-Cell culture (THP1human monocytes)-IC_50_ for drugs: 63.1, >100 or 98.3 μg/mL, respectively	Active [116]
	*C. cuspidatum *Planch. ex Benth.	MeOH, MeOH/H_2_O (50:50) or MeCl_2 _ext. of stem barks	*In vitro*-Cell culture (THP1 human monocytes)-IC_50_ for drugs: >100, >100 or 25.3 μg/mL, respectively	Active [116]
	*C. duarteanum* Cambess.	EtOH (95%) ext. of dried leaf	*In vitro*-Cell culture (KB cells)—Concentration not cited	Active [153]
		EtOH (95%) ext. of dried root	*In vitro*-Cell culture (KB cells)—Concentration not cited	Active [153]
		EtOH (95%) ext. of dried stem	*In vitro*-Cell culture (KB cells)—Concentration not cited	Active [153]
	*C. fragrans* F. Hoffm.	MeOH ext. of dried leaf or dried root	Cell culture (T24 bladder, , HeLa cervical or MCF7 breast cancer)—Concentration for drugs: 25 µg/mL	Active [15]
	*C. fruticosum* (Loefl.) Stuntz	EtOAc ext	*In vitro*-Cell culture (CA-9KB)—ED_50_ for drug: 6.5 μg/mL	Active [154]
		H_2_O ext	*In vitro*-Cell culture (CA-9KB)—ED_50_ for drug: 10.0 μg/mL	Active [154]
		Type ext. not stated	*In vitro*-Cell culture (CA-9KB)—Dose for drug: >100 μg/mL	Inactive [154]
		Hexane ext.	*In vitro*-Cell culture (CA-9KB)—ED_50_ for drug: 11.0 μg/mL	Active [154]
	*C. hereroense *Schinz	MeOH ext. of dried stem bark	Cell culture (T24 bladder, HeLa cervical or MCF7 breast cancer)—Concentration for drug: 25µg/mL	Active [15]
	*C. micranthum *G. Don	MeOH ext. of dried leaf	*In vitro*-Cell culture (human monocytes-THP1 cells)—Concengration for drug: >25.0 μg/mL	Inactive [61]
		MeOH ext. of dried leaf or dried root	*In vitro*-Cell culture (T24 bladder, HeLa cervical or MCF7 breast cancer)—Concentration for drugs: 25 µg/mL	Active [15]
	*C. nigricans *Lepr.	MeOH ext. of fresh leaf	*In vitro*-Cell culture (U-373 MG human astrocytoma cells)—IC_50_ for drug: 41.0 μg/mL	Active [155]
			*In vitro*-Cell culture (HCT-15 colon human cells)—IC_50_ for drug: 41.0 μg/mL	Active [155]
			*In vitro*-Cell culture (A549 cancer cells)—IC_50_ for drug: 41.0 μg /mL	Active [155]
			*In vitro*-Cell culture (J82 human urothelial cells)—IC_50_ for drug: 41.0 μg/mL	Active [155]
	*C. ovalifolium *Roxb. var. *cooperi*	EtOH-H_2_O (1:1) ext. of aerial parts	*In vitro*-Cell culture (CA-9KB cells)—Dose for drug: >20.0 μg/mL	Inactive [152]
	*C. padoides* Engl. & Diels	MeOH ext. of dried stem bark	*In vitro*-Cell culture (T24 bladder, HeLa cervical or MCF7 breast cancer)—Concentration for drug: 25 µg/mL	Active [15]
		MeOH ext. of dried root	*In vitro*-Cell culture (T24 bladder, HeLa cervical or MCF7 breast cancer)—Concentration for drug: 25 µg/mL	Active [15]
	*C. psidioides* Welw.	MeOH ext. of dried stem bark	*In vitro*-Cell culture (T24 bladder, HeLa cervical or MCF7 breast cancer)—Concentration for drug: 25 µg/mL	Active [15]
	*C. zeyheri* Sond.	MeOH ext. of dried fruit	*In vitro*-Cell culture (T24 bladder, HeLa cervical or MCF7 breast cancer)—Concentration for drug: 25 µg/mL	Active [15]
		MeOH ext. of dried root	*In vitro*-Cell culture (T24 bladder, HeLa cervical or MCF7 breast cancer)—Concentration for drug: 25 µg/mL	Active [15]
		MeCl_2_ ext. of dried leaf	*In vitro*-Cell culture (Renal TK10, Breast MCF7 or Melanoma UACC62 cancer)—IC_50_ for drug: 15.00, 28.21 or 10.33 mg/mL, respectively	Active [156]
	*C. erythrophyllum* (Burch) Sond	MeOH ext. of dried wood	*In vitro*-DNA damage assay-Cell culture Ycp (gal) or pRAD52 (glu)—IC_50_ for drug: 4.0 or 15 μg/mL, respectively	Active [91]
			*In vitro*-DNA damage assay-Cell culture pRAD52 (gal)—IC_50_ for drug: >100 μg/mL	Inactive [91]
		MeCl_2_ ext. of dried wood	*In vitro*-DNA damage assay-Cell culture Ycp (gal), pRAD52 (gal), pRAD52 (glu), phTOP1 (gal) or phTOP1 (glu)—IC_50_ for drug: 2.0, 34.0, 31.0, 3.3 or 4.3 μg/mL, respectively	Active [91]
			*In vitro*-DNA damage by agar diffusion assay (RS188-WT erg6 or RS321-Rad52.erg6.top1)—IC_50_ for drug: 73.7 or 5.9 μg/mL, respectively	Active [91]
			*In vitro*-DNA damage by agar diffusion assay (RS322-Rad52.erg6)—IC_50_ for drug: >100 μg/mL	Inactive [91]
		EtOAc soluble fraction of dried wood	*In vitro*-DNA damage assay-Cell culture Ycp (gal) or pRAD52 (glu)—IC_50_ for drug: 4.0 or 12 μg/mL, respectively	Active [91]
			*In vitro*-DNA damage assay-Cell culture pRAD52 (gal)—IC_50_ for drug: >100 μg/mL	Inactive [91]
Brine shrimp lethality				
	*C. aculeatum* Vent.	MeCl_2_, MeOH and H_2_O ext. of dried leaf, dried root bark or dried stem	*In vitro*-Toxicity bioassay with *Artemia salina* L.—Concentration for all drugs: 500.0 μg/mL	Inactive [122]
	*C. micranthum *G. Don	EtOH (100%) ext. of dried leaf	*In vitro*-Toxicity bioassay with *A. salina* L.—LC_50_ for drug: 112.0 μg/mL	Active [56]
		CHCl_3_ or H_2_O ext. of dried leaf	*n vitro*-Toxicity bioassay with *A. salina* L.—LC_50_ for drugs: 492.0 or 634.0 μg/mL, respectively	Inactive [56]
		EtOH (100%) ext. of dried bark	*In vitro*-Toxicity bioassay with *A. salina* L.—LC_50_ for drug: 432.0 μg/mL	Inactive [56]
	*C. zeyheri *Sond	MeOH ext. of dried root	*In vitro*-Toxicity bioassay with *A. salina* L.—Concentration for all drugs: >0.1 mg/mL	Inactive [157]
Molluscicidal				
	*C. aculeatum* Vent.	MeCl_2_, MeOH or H_2_O ext. of dried leaf, dried root or dried stem	*In vitro*-Toxicity bioassay with *Biomphalaria glabrata*—Concentration for all drugs: 400.0 ppm	Inactive [122]
	*C. dolichopetalum *Gils ex Engl.	MeOH ext. of dried leaf	*In vitro-*Toxicity bioassay with *Bulinus globosus* snail—Concentration for drug: 100.0 ppm	Inactive [158]
	*C. ghasalense *Engl. & Diels	MeOH ext. of dried fruit or dried leaf	*In vitro*-Toxicity bioassay with *B. globosus* snail—Concentration for all drugs: 100.0 ppm	Inactive [158]
		MeOH ext. of dried root or dried stem	*In vitro-*Toxicity bioassay with *B. globosus* snail—Concentration for all drugs: 100.0 ppm	Active [158]
		MeOH ext. of dried stem	*In vitro*-Toxicity bioassay with *B. globosus* snail—Concentration for drug: 100.0 ppm	Active [159]
	*C. glutinosum *Perrot. ex DC	MeOH ext. of dried fruit, dried root or dried stem	*In vitro*-Toxicity bioassay with *B. globosus* snail—Concentration for all drugs: 100.0 ppm	Inactive [158]
	*C. leprosum* Mart.	EtOH (95%) or H_2_O ext. of dried stem bark	*In vitro*-Toxicity bioassay with *B. glabrata* or *B. straminea*—Concentration for all drugs: 1,000 ppm	Active [160]
	*C. micranthum *G. Don	MeOH ext. of dried leaf	*In vitro-*Toxicity bioassay with *B. globosus* snail—Concentration for drug: 100.0 ppm	Inactive [158]
	*C. molle *R. Br. ex G. Don	H_2_O ext. of dried leaf	*In vitro-*Toxicity bioassay with *Biomphalaria pfeifferi*—Concentration for drug: 1:1,000 (v:v)	Active [75]
Toxicity on mammals				
	*C. decandrum *Roxb. (DC)	EtOH 50% ext. of entire plant	Lethal dose 50% (LD_50_) in mice—LD_50_ for drug: 1.0 mg/kg (*i.p.*)	Active [161]
	*C. dolichopetalum* Gils ex Engl.	EtOH (70%) ext. of dried root bark	LD_50_ in rats—LD_50_ for drug: 246.0 mg/kg (*i.p.*)	Active [97]
	*C. hypopilium*	MeOH ext.	LD_50_ in mice—LD_50_ for drug: 2.3 mg/kg (*i.v.*)	Active [150]
	*C. leprosum *Mart.	EtOH (70%) ext. of dried stem bark	LD_50_ in mice—LD_50_ for drug: 4,722 mg/kg (*p.o.*)	Active [143]
	*C. nanum* Ham. *ex *D. Don.	EtOH-H_2_O (1:1) ext. of dried entire plant	LD_50_ in mice—LD_50_ for drug: 500.0 mg/kg (*i.p.*)	Active [162]
	*C. nigricans *Lepr.	MeOH ext. of seed	Lethal dose 50% (LD_50_) in mice—LD_50_ for drug: 580.0 mg/kg (*i.v.*)	Active [150]
	*C. ovalifolium *Roxb.var. *cooperi*	EtOH-H_2_O (1:1) ext. of aerial parts	Lethal dose 50% (LD_50_) in mice—LD_50_ for drug: 500.0 mg/kg (*i.p*)	Active [152]
	*C. racemosum* P. Beauv	Hot H_2_O or EtOH (95%) ext. of dried leaf	Lethal dose 50% (LD_50_) in mice—LD_50_ for drug: 17.78 mL/kg (*i.p*)	Active [151]
	*C. sokodense* Eng.	MeOH ext. of seed	Lethal dose 50% (LD_50_) in mice—LD_50_ for drug: 700.0 mg/kg (*i.v.*)	Active [150]
	*C. verticillatum* Engl. & Diels	MeOH ext. of seed	Lethal dose 50% (LD_50_) in mice—LD_50_ for drug: 800.0 mg/kg (*i.v.*)	Active [150]
Antihepatotoxicity				
	*C. dolichopetalum *Gils ex Engl.	EtOH (95%) ext. of fresh root bark	Paracetamol-induced hepatotoxicity in rat—Dose for drug: 100.0 mg/kg (*p.o.*)	Active [95]
	*C. quadrangulare *Kurz.	MeOH ext. of dried seed	D-Galactosamine (D-GalN)/tumor necrosis factor-alpha(TNF-alpha)-induced hepatotoxicity in mice—IC_50_ for drug: 56.4 μg /mL	Active [101]
		MeOH/H_2_O (1:1) or H_2_O ext. of dried seed	D-Galactosamine (D-GalN)/tumor necrosis factor-alpha(TNF-alpha)-induced hepatotoxicity in mice —Concentration for drug: 100.0 μg/mL	Inactive [101]
		H_2_O soluble fraction of dried seed	D-Galactosamine (D-GalN)/tumor necrosis factor-alpha(TNF-alpha)-induced hepatotoxicity in mice —Concentration for drug: IC_50_ 39.3 μg/mL	Active [101]
		MeOH soluble fraction of dried seed	D-Galactosamine (D-GalN)/tumor necrosis factor-alpha(TNF-alpha)-induced hepatotoxicity in mice—Concentration for drug: 42.1 μg/mL	Inactive [101]
Abortifacient				
	*C. glutinosum *Perrot. ex DC	Decoction of leaf	*In vitro*-Inhibit hepatitis B virus antigen (HBsAg)—Concentration for drug: 100–500 ng/mL	Active [147]
	*C. racemosum* P. Beauv	Hot H_2_O ext. of dried leaf	Abortion in 7 days after oral administration of 10 g/mL in pregnant guinea pig	Active [151]
Embryotoxic				
	*C. molle *R. Br. ex G. Don	Hot H_2_O ext. of dried entire plant	Rats treated with extract in dose of 10.0 mg/kg (*p.o.*)	Inactive [79]
*Gastrintestinal activity*				
Gastric antiulcer				
	*C. dolichopetalum*	EtOH (70%) ext. of dried root	Pyloric ligation together with histamine-induced ulcers and gastric secretions in rats—Dose for drug: 400.0 mg/kg (*p.o.*)	Active [93]
		EtOH (16%) ext. of dried root	Indomethacin and cold strees-induced ulcers in guinea pig—Dose for drug: 100.0 mg/kg (*p.o.*)	Active [94]
	*C. duarteanum* Cambess	EtOH or Hexane ext. of dried leaf	HCl/Ethanol, piroxican or immobilization-cold strees-induced ulcers in mice—Dose for drug: 62.5, 125, 250 and 500 mg/kg (*p.o.*)	Active [163]
	*C. leprosum *Mart. & Eiche	EtOH ext. of dried stem bark	Ethanol or Indomethacin induced gastric ulcer in rats—Doses for drug: 60, 125 and 250 mg/kg (*p.o.*)	Active [164]
Gastric emptying				
	*C. dolichopetalum *Gils ex Engl.	EtOH (70%) ext. of dried root	Delayed gastric emptying in rat—Dose for drug: 400.0 mg/kg (*p.o.*)	Active [93]
Antispasmodic				
	*C. ovalifolium var.cooperi*	EtOH-H_2_O (1:1) ext. of aerial parts	Ach and histamine-induced contractions in guinea pig ileum—Concentration not cited	Active [152]
	*C. racemosum* P. Beauv	Hot H_2_O ext. of dried leaf	Ach, nicotine or histamine-induced contractions in guinea pig ileum—Concentration for drug:1.0g/mL	Active [151]
			Spontaneous contractions in rabbit jejunum blocked by DHE and propranolol—Concentration for drug: 0.2–1 g/mL	Inactive [151]
	*C. dolichopetalum *Gils ex Engl.	EtOH (70%) ext. of dried root	Ach or histamine-induced contractions in guinea pig ileum—Concentration for drug: 0.24 μg/mL	Active [93]
			Ach or histamine-induced contractions in guinea pig ileum—Concentration for drug: 10 μg/mL	Active [94]
			Relaxation effect in guinea pig ileum—EC_50_ for drug: 2.65 mg/mL	Active [94]
*Geniturinary activity*				
	*C*. *erythrophyllum* (Burch.) Sond.	H_2_O or EtOH (95%) ext. of dried leaf	*In vitro*-Radioactivity of cyclooxygenase (prepared from sheep seminal vesicle microsomal fractions)—Concentration for drugs: 20.0 mg/mL or 2.5 mg/mL, respectively	Active [92]
		H_2_O or EtOH (95%) ext. of dried leaf	Ach-induced contractility uterine in guinea pig—Dose for drugs: 10 mg/mL	Active [92]
			Oxytocin-induced contractility uterine in guinea pig—Dose for drugs: 10 mg/mL	Inactive [92]
		Hot H_2_O ext. of dried branch and leaf	*In vitro*-Contractions of uterus isolated from rat—Concentration not cited	Active [165]
	*C. kraussii* Hochst.	Hot H_2_O ext. of dried root	*In vitro*-Contractions of uterus isolated from rat—Concentration not cited	Active [165]
	*C. nanum* Ham. ex. D. Don.	EtOH/H_2_O (1:1) ext. of dried entire plant	Spermicidal effect in rat—Concentration not cited	Inactive [162]
	*C. platypetalum* Sond.	H_2_O or EtOH (95%) ext. of dried leaf	*In vitro*-Radioactivity of cyclooxygenase (prepared from sheep seminal vesicle microsomal fractions)—Concentration for drugs: 20.0 mg/mL or 2.5 mg/mL, respectively	Active [92]
			Ach or oxytocin-induced contractility uterine in guinea pig—Dose for drugs: 10 mg/mL	Inactive [92]
	*C. racemosum* P. Beauv	Hot H_2_O ext. of dried leaf	*In vitro*-Contractions in guinea pig gravid and non-gravid uterus blocked by hydergine—Concentration for drug: 1–2 g/mL	Inactive [151]
			Ext. induced spontaneus contractions in guinea pig vas deferens—Concentration for drug: 0.5 g/mL	Active [151]
	*C. zeyheri *Sond.	H_2_O or EtOH (95%) ext. of dried bark	Radioactivity of cyclooxygenase (prepared from sheep seminal vesicle microsomal fractions)—Concentration for drugs: 20.0 mg/mL or 2.5 mg/mL, respectively	Active [92]
			Ach or oxytocin-induced contractility uterine in guinea pig—Dose for drugs: 10 mg/mL	Inactive [92]

*i.p. *= intraperitoneal; *p.o*. = oral; *i.v*. = intravenous; EtOH ext. = ethanolic extract; H_2_O ext. = aqueous extract; MeOH ext. = methanolic extract; EtOAc ext. = ethyl acetate extract; CHCl_3_ ext. = chloroformic extract; CCl_4_ ext. = carbon tetrachloride extract; MeCl_2_ ext. = dichloromethane extract; EtOH/H_2_O ext. = crude aqueous/alcoholic extract; MeOH/H_2_O ext. = aqueous/methanolic extract; CHCl_3_/MeOH ext. = chloroformic and methanolic extract; MeOH/MeCl_2_ ext. = methanolic/dichloromethane extract; Pet ether ext. = Petroleum ether extract. Ach = Acethylcholine; DHE = Dihydroergotamine; ACE = Angiotensin converting enzyme.

## 4. Conclusions

The research papers cited in this review contribute to justifying the traditional use of the genus *Combretum* for the treatment of various health problems. This genus presents itself as a promising new scientific research topic to investigate the pharmacological potential of the extracts, fractions and compounds isolated from plant species of this genus.

We see that there is a need for further studies on the standardization or chemical characterization of the extracts used and for other more detailed phytochemical studies. With respect to pharmacological studies, there is an increasing need for further *in vivo* investigations of toxicity and biological activities, as well as for insights into the possible mechanisms involved. Therefore, new research findings could lead to greater safety and benefits to people who use these species to treat diseases, contributing to a better access to health care and thereby a better quality of life. 
